# Tailoring Gene Transfer Efficacy through the Arrangement of Cationic and Anionic Blocks in Triblock Copolymer Micelles

**DOI:** 10.1021/acsmacrolett.3c00633

**Published:** 2024-01-17

**Authors:** Katharina Leer, Liên S. Reichel, Mara Wilhelmi, Johannes C. Brendel, Anja Traeger

**Affiliations:** † Laboratory of Organic and Macromolecular Chemistry, Friedrich Schiller University Jena, Humboldtstrasse 10, 07743 Jena, Germany; ‡ Jena Center for Soft Matter, Friedrich Schiller University Jena, Philosophenweg 7, 07743 Jena, Germany

## Abstract

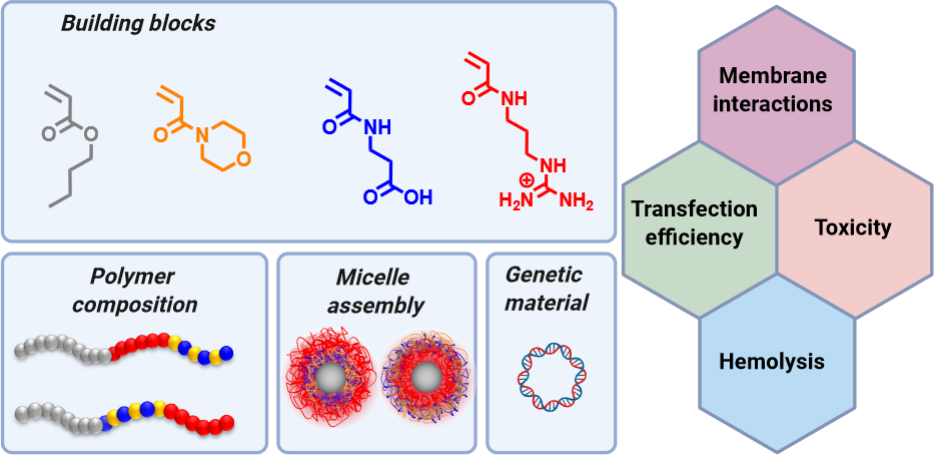

The arrangement of charged segments in triblock copolymer micelles affects the gene delivery potential of polymeric micelles and can increase the level of gene expression when an anionic segment is incorporated in the outer shell. Triblock copolymers were synthesized by RAFT polymerzation with narrow molar mass distributions and assembled into micelles with a hydrophobic core from poly(*n*-butyl acrylate). The ionic shell contained either (i) an anionic segment followed by a cationic segment (**HAC** micelles) or (ii) a cationic block followed by an anionic block (**HCA** micelles). The pH-responsive anionic block contained 2-carboxyethyl acrylamide (CEAm), while the cationic block comprised 3-guanidinopropyl acrylamide (GPAm). Increasing the molar content of CEAm in **HAC** and **HCA** micelles from 6 to 13 mol % improved cytocompatibility and the endosomal escape property, while the **HCA** micelle with the highest mol % of anionic charges in the outer shell exhibited the highest gene expression. It became evident that improved membrane interaction of the best performing **HCA** micelle contributed to achieving high gene expression.

Research in the field of nonviral gene therapy gained greater awareness and boost with the successful development of lipid-based vaccines for COVID-19.^1,2^ Despite the success of lipid nanoparticles in RNA delivery, challenges remain for more complex applications and other genetic materials, which demand further research on smart and stable delivery systems to fulfill the promise of gene therapy. Nonviral nanocarriers can also be based on polymers, which can be conveniently tailored in their composition and morphology as more versatile synthetic approaches are developed.^3−7^ They can form stable complexes (polyplexes) with the negatively charged genetic material to promote cellular uptake and endosomal release, enabling high gene expression and low toxicity.^8^ Among them, amphiphilic block copolymers containing hydrophobic and hydrophilic segments are attractive architectures,^9,10^ which can assemble into core–shell micelles in an aqueous system.^11,12^ If cationic charges are present in the shell they can form so-called micelleplexes by ionic interactions with genetic material, which exhibit increased colloidal stability and improved gene expression.^13−15^ Due to their high positive charge density, cytotoxic effects were also observed for polymeric micelles, albeit not as severe as for linear and branched polymers.^16,17^ The incorporation of hydrophilic polymers, such as PEG (poly(ethylene glycol)),^18^ poly(*N*-acryloyl morpholine) (PNAM),^19^ poly(2-oxazoline),^20^ or polysarcosine,^21^ is the most common approach to attenuate the toxicity of cationic polymers. These “stealth” polymers induce a hydration layer, which reduces interactions with serum proteins and increases circulation time in blood.^22^ However, the improved biosafety profile is often accompanied by a reduced efficiency, known as the toxicity-efficiency dilemma.^18,23^ Recent studies show that this dilemma can be circumvented by inserting negatively charged functionalities into the polymeric carrier instead, which partially compensate the positive charges.^4,24−26^ Anionic groups can be integrated in the form of a polymer either (i) electrostatically as a coating of a cationic polyplex or (ii) by covalent incorporation into a (block) copolymer structure.^27−30^ The first approach is more straightforward in terms of the synthesis of the polymers, but requires optimization of the formulation conditions (mixing order, ratio of opposite charges).^30−33^ By contrast, the challenge of the second approach is the controlled synthesis of (block) copolymers containing positively and negatively charged functionalities. To date, only a few studies exist, where anionic functionalities have been covalently incorporated into polymeric micelles for gene delivery.^26,34,35^ Thus, the potential of anionic charges in nanocarrier systems has not yet been fully exploited due to limited knowledge of advantageous compositions and monomer sequences.

Therefore, a structure–property study was performed to ascertain the transfection efficiency of triblock micelles with an ionic/hydrophilic shell containing ether (i) an anionic block followed by a cationic block or vice versa (ii) a cationic block followed by an anionic block. The core-forming hydrophobic block was based on poly(*n*-butyl acrylate) (P*n*BA), while the anionic copolymer block consisted of 2-carboxyethyl acrylamide (CEAm) and hydrophilic NAM and the cationic block contained 3-guanidinopropyl acrylamide (GPAm). The combination of a pH-dependent anionic group with a pH-independent cationic group represents a novel approach to gene therapy, deviating from the conventional use of pH-dependent cationic groups. The formed micelles and micelleplexes were characterized regarding their physicochemical and biological behavior, and it was clearly observed that the anionic outer block provides advantages for their application in gene delivery.

For the assembly of micelles, two sets of triblock copolymers were synthesized by sequential reversible addition–fragmentation chain transfer (RAFT) polymerization (Figure 1A). For the multiblock synthesis, the chain transfer agent (CTA) (propanoic acid)yl butyl trithiocarbonate (PABTC) was used, since it has optimal addition and fragmentation rates for the polymerization of acrylates and acrylamides.^36,37^ The synthesis of multiblock copolymers with controlled molar masses can be challenging due to the accumulation of dead chains after each consecutive chain extension, leading to high dispersity (*Đ*).^38^ This highlights the importance of maintaining a high proportion of chains with the thiocarbonylthio group throughout the polymerization. Since acrylates and acrylamides possess high propagation rate coefficients, the initiator concentration can be reduced while still achieving high monomer conversions, and thus, the fraction of living chains remains high.^36,39^ Furthermore, the azoinitiator V-65B was chosen, since it generates radicals at an optimal rate at lower temperatures (10 h half-time decomposition temperature of 51 °C in toluene).^40^ First, *n*BA was polymerized at 50 °C in 1,4-dioxane to obtain P(*n*BA) as the first hydrophobic block (**H**) and macroCTA (Figure 1A). For the first set of triblock copolymers, P(*n*BA) was chain extended with *tert*-butyl-protected CEAm (CEAm^*t*B^) and NAM as the anionic block (**A**) obtaining P[(*n*BA)-*b*-(CEAm^*t*B^-*co*-NAM)], followed by a chain extension with diBoc-protected GPAm (GPAm^diBoc^) as the cationic block (**C**), yielding P[(*n*BA)-*b*-(CEAm^*t*B^-*co*-NAM)-*b*-(GPAm^diBoc^)] (**HAC**^**pro**^, protected variant). For the second set of triblock copolymers, P(*n*BA) was first chain extended with GPAm^diBoc^, followed by a chain extension with CEAm^*t*B^ and NAM, obtaining P[(*n*BA)-*b*-(GPAm^diBoc^)-*b*-(CEAm^*t*B^-*co*-NAM)] (**HCA**^**pro**^, protected variant). The two sets each consisted of three triblock copolymers with a comparable degree of polymerization (DP) of the hydrophobic block (**H**, DP ≈ 80) and either (i) an anionic middle block followed by a cationic outer block or (ii) a cationic middle block followed by an anionic outer block (Table 1). Preliminary experiments with block copolymers showed that a molar amount of GPAm greater than 30 mol % and low amounts of CEAm are needed to achieve high transfection efficiencies, using NAM as a “stealth” moiety and for increased colloidal stability.^41^ Therefore, the amount of cationic GPAm (**C**) was varied between 30 and 37 mol %, while the amount of anionic CEAm (**A**) ranged from 6 to 14 mol %, which is shown in the bar chart of Figure 1B. Numbers after hyphen (**HAC**-g/c and **HCA**-g/c) represent the mol % of GPAm (g) and the mol % of CEAm (c).

**Figure 1:**
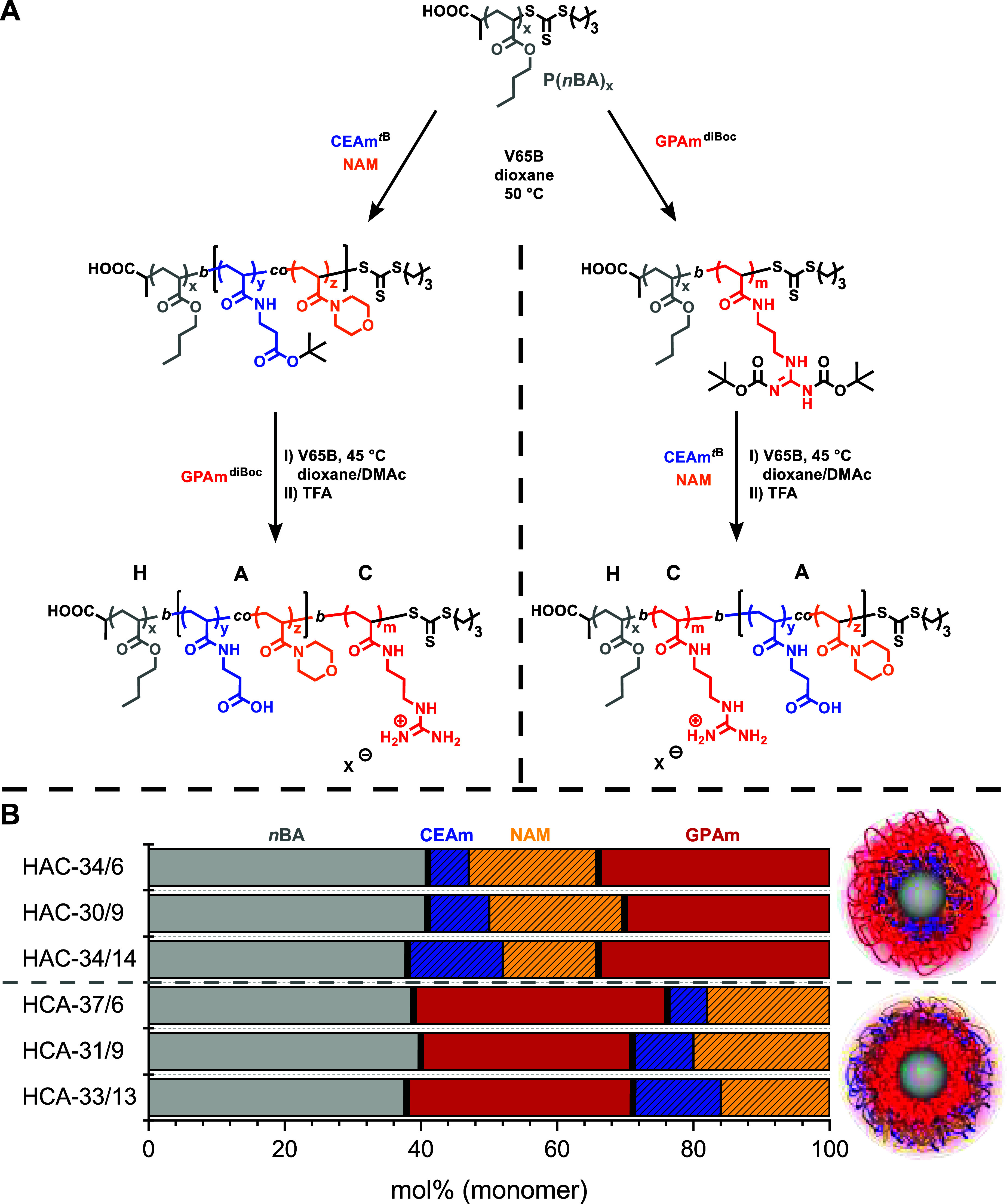
(A) Synthesis of the triblock copolymers **HAC**-g/c and **HCA**-g/c by RAFT polymerization and subsequent deprotection (X^–^:F_3_CCOO^–^). (B) Compositions of the six triblock copolymers are pictured in a bar diagram with the respective *n*BA, NAM, GPAm, and CEAm content in mol %.

**Table 1:** Overview of the Composition and Characterization of the Protected Triblock Copolymers.

polymer code-g/c^a^	composition^b^	*M*_n,theo_^c^ (kg mol^–1^)	*M*_n,SEC_^d^ (kg mol^–1^)	*Đ*^d^
**HAC**^**pro**^-34/6	P[(*n*BA)_78_-*b*-(CEAm^*t*B^_11_-*co*-NAM_35_)-*b*-(GPAm^diBoc^)_65_]	41.5	42.2	1.20
**HAC**^**pro**^-30/9	P[(*n*BA)_78_-*b*-(CEAm^*t*B^_17_-*co*-NAM_37_)-*b*-(GPAm^diBoc^)_56]_	39.6	36.7	1.22
**HAC**^**pro**^-34/14	P[(*n*BA)_80_-*b*-(CEAm^*t*B^_30_-*co*-NAM_30_)-*b*-(GPAm^diBoc^)_72_]	47.4	45.9	1.24
**HCA**^**pro**^-37/6	P[(*n*BA)_78_-*b*-(GPAm^diBoc^)_73_-*b*-(CEAm^*t*B^_11_-*co*-NAM_36_)]	44.6	50.1	1.29
**HCA**^**pro**^-31/9	P[(*n*BA)_78_-*b*-(GPAm^diBoc^)_61_-*b*-(CEAm^*t*B^_17_-*co*-NAM_39_)]	41.7	48.5	1.21
**HCA**^**pro**^-33/13	P[(*n*BA)_80_-*b*-(GPAm^diBoc^)_69_-*b*-(CEAm^*t*B^_27_-*co*-NAM_33_)]	46.1	54.2	1.19

a g: mol % of GPAm, c: mol % of CEAm.

b Numbers were determined via ^1^H NMR spectroscopy and represent the DP of each monomer.

c Calculated using eq S2.

d Determined via SEC (eluent: DMAc + 0.21% LiCl; PMMA standard).

P(*n*BA), the diblock and final protected triblock copolymers were analyzed by size exclusion chromatography (SEC) to determine the experimental number-average molar masses (*M*_n,SEC_) and *Đ* (Table 1 and Figure S8 and Table S6, SI). Exemplary, the SEC traces of **HAC**^**pro**^-30/9 (cationic outer block), **HCA**^**pro**^-31/9 (anionic outer block), and their precursors are shown in Figure 2. P(*n*BA)_78_ revealing a narrow molar mass distribution with *Đ* = 1.07, which shifted to higher molar masses after chain extension, while maintaining their monomodal and narrow character (Figure 2A). After the second chain extension with GPAm^diBoc^, the population shifted to higher molar masses, revealing a tailing towards lower molar masses. This might be due to dead polymer chains caused by recombination throughout the block extensions and precursor chains that failed to reinitiate, resulting in a broadened molar mass distribution (*Đ* = 1.22).^42^ For the synthesis of **HCA**^**pro**^-31/9, the first chain extension with GPAm^diBoc^ led to a broadened population with a slight tailing towards lower molar masses (*Đ* = 1.24; Figure 2B). After the subsequent chain extension with CEAm^*t*B^ and NAM, the population shifted to higher molar masses and a more narrow dispersity (*Đ* = 1.21). The differences between experimental and theoretical number-average molar masses can be attributed to differences in hydrodynamic volume from the PMMA standards. Furthermore, the kinetics of the copolymerization of CEAm^*t*B^ and NAM were investigated by ^1^H NMR spectroscopy and SEC (Figures S9–S11, SI), indicating a controlled copolymerization process, which resulted in a slightly gradient structure due to the more reactive NAM. Both triblock copolymers were deprotected with trifluoroacetic acid (TFA) to expose the guanidinium and carboxy group. The deprotection was successful, since the peak of the Boc-/*tert*-butyl group at 1.5 ppm vanished, obtaining the final deprotected **HAC**-g/c and **HCA**-g/c polymers (Figures S12–S14, SI).

**Figure 2:**
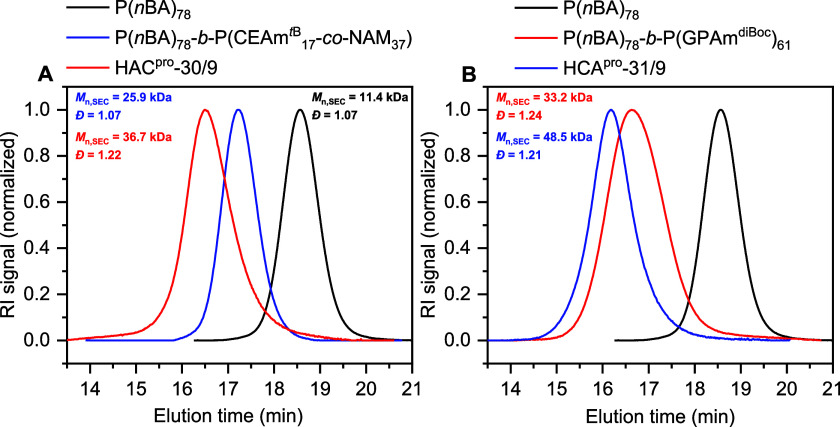
SEC traces of P(*n*BA)_78_, precursors, and (A) **HAC**^**pro**^-30/9 and (B) **HCA**^**pro**^-31/9 (eluent: DMAc + 0.21% LiCl, PMMA-calibration).

The triblock copolymers were assembled into micelles using the solvent exchange approach,^12^ where the polymers are first dissolved in a mixture of tetrahydrofuran/methanol (THF/MeOH 80/20 v/v%). Ultrapure water was added slowly, followed by dialysis against 20 mM sodium acetate buffer (pH 5), generating micelles with a hydrophobic P(*n*BA) core and a hydrophilic shell. Since the guanidinium group is fully charged independent of the used pH-value (apparent p*K*_a_ > 12),^40^ the pH-responsive element at physiological pH is the anionic block containing CEAm and NAM (apparent p*K*_a_(PCEAm) ≈ 5.1).^31^ At a pH of 5, only about half of the carboxy groups are charged, while at pH 7.4 almost all are charged (92%). Therefore, initial attempts to formulate the triblock micelles at physiological pH values failed and led to precipitation. At these pH values, both polymer blocks are highly charged and interact strongly, which might destabilize the triblock micelle solutions at the given elevated concentrations.

Size investigations by dynamic light scattering (DLS) revealed a similar *Z*-average value of hydrodynamic size for all six triblock micelles under acidic conditions ranging from 25 to 36 nm (Figure 3). The triblock micelles with a cationic outer block (**HAC**-34/6, -30/9, -34/14) showed monomodal size distributions with narrow polydispersity indices (PDIs) ranging from 0.1 to 0.2 (Figure 3, Figure S15, SI). In contrast, the triblock micelles with an anionic outer block (**HCA**-37/6, -31/9, -33/13) showed a bimodal intensity-weighted size distribution with increased PDIs (0.25–0.34), which was most prominent for **HCA**-33/13 (Figure S16, SI). Since cationic and anionic moieties were combined within the shell of the triblock micelles, an interpolyelectrolyte complex (IPEC) was expected to be formed in the shell.^43^ Thus, the micelles with a cationic outer block (**HAC**) formed more uniform micelles compared to the **HCA** micelles, containing CEAm and NAM in the outer shell.

**Figure 3:**
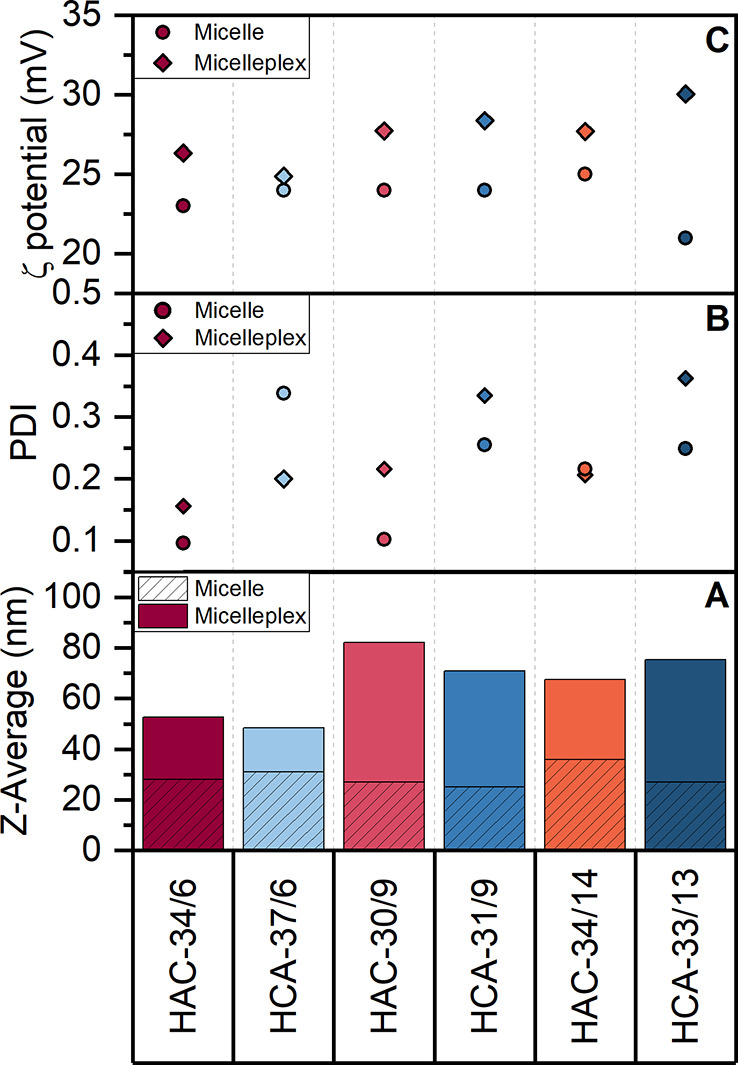
(A) *Z*-Average, (B) PDI, and (C) ζ-potential of the triblock copolymer micelles and micelleplexes measured by DLS. Details can be found in the Supporting Information (Figures S15–S18).

With the different micelles at hand, we now investigated the formation of micelleplexes using pDNA. In preliminary experiments, N*/P ratios (molar ratios of protonatable nitrogen atoms to phosphates of pDNA) of 20 and 3 μg mL^–1^ pDNA were found to be optimal to achieve high transfection efficiency and low toxicity. The complexation with the genetic material resulted in micelleplexes with sizes between 49 nm and 82 nm, which is still suitable for efficient cellular uptake by endocytosis, but larger than the initial micelles.^44^ The intensity-weighted size distributions were monomodal for most of the micelleplexes with a maximum PDI of 0.36 for **HCA**-33/13 (Figures S17 and S18, SI). The ζ-potentials of all micelles/micelleplexes were above 20 mV independent of the sequence, which can be attributed to the excess of protonated amines due to the higher molar ratio of cationic to anionic charges in the triblock micelles and the excess of polymer micelles used for the formulation.

The cytotoxicity profiles of the polymer library were investigated via the PrestoBlue assay. Based on ISO10993–5, the mouse fibroblast cell line L929 was used. Figure 4A shows a decrease in metabolic activity with an increasing polymer concentration. The incorporation of stealth and anionic functionalities showed a positive effect on the cytocompatibility of the micelles in comparison to the cationic homopolymer poly(GPAm)_71_ (Gua 100), which served as control. It should be noted that the cytocompatibility improved with increasing mol % content of CEAm, whereas the sequence had lower impact (**HCA**-33/13 vs **HAC**-34/14, LC_50_ in Table S7).

**Figure 4:**
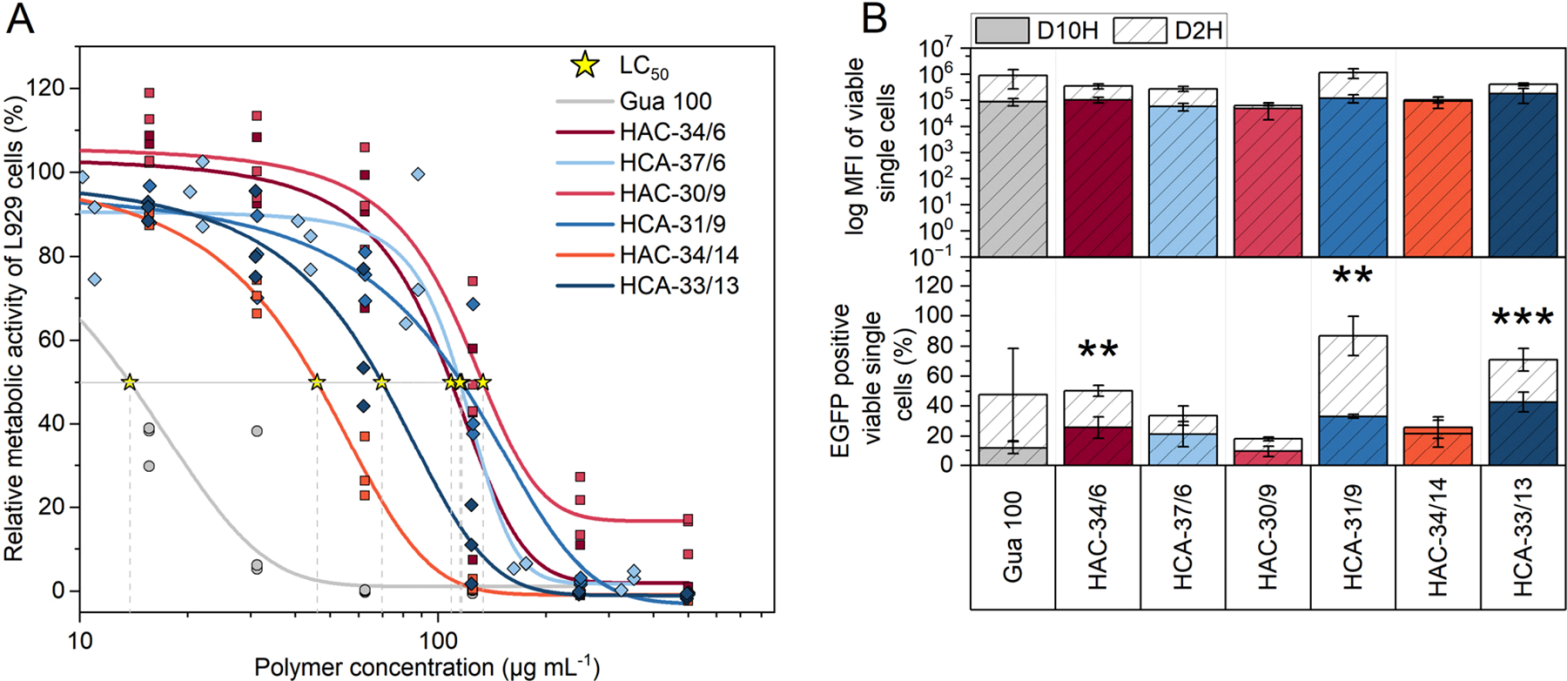
(A) PrestoBlue assay was performed over 24 h using D10H in the L929 cell line. (B) Transfection efficiencies were investigated in D2H (dashed bars) and D10H (colored bars) for 24 + 24 h in the HEK293T cell line at N*/*P* 20 using 3 μg mL^–1^ of EGFP expressing pDNA. Values represent mean ± SD (*n* ≥ 3). Significances are illustrated as p* > 0.05, p** > 0.01, p*** > 0.001.

At a physiological pH value, the guanidinium group is positively charged and tends to interact with serum proteins. Therefore, a serum-reduced medium D2H (Dulbecco’s modified eagle medium (DMEM) with 2% fetal bovine serum (FBS) and 10 mM 2-[4-(2-hydroxyethyl)piperazin-1-yl]ethanesulfonic acid (HEPES) buffer) was used for the transfection assays. To assess the effect of the triblock composition on gene delivery, additional transfection was performed in D10H (10% FBS), which is closer to physiological conditions. Due to the low serum concentration in D2H, higher transfection efficiencies could generally be achieved in comparison to D10H (Figure 4B: plot below, dashed bars vs colored bars).

**Figure 5:**
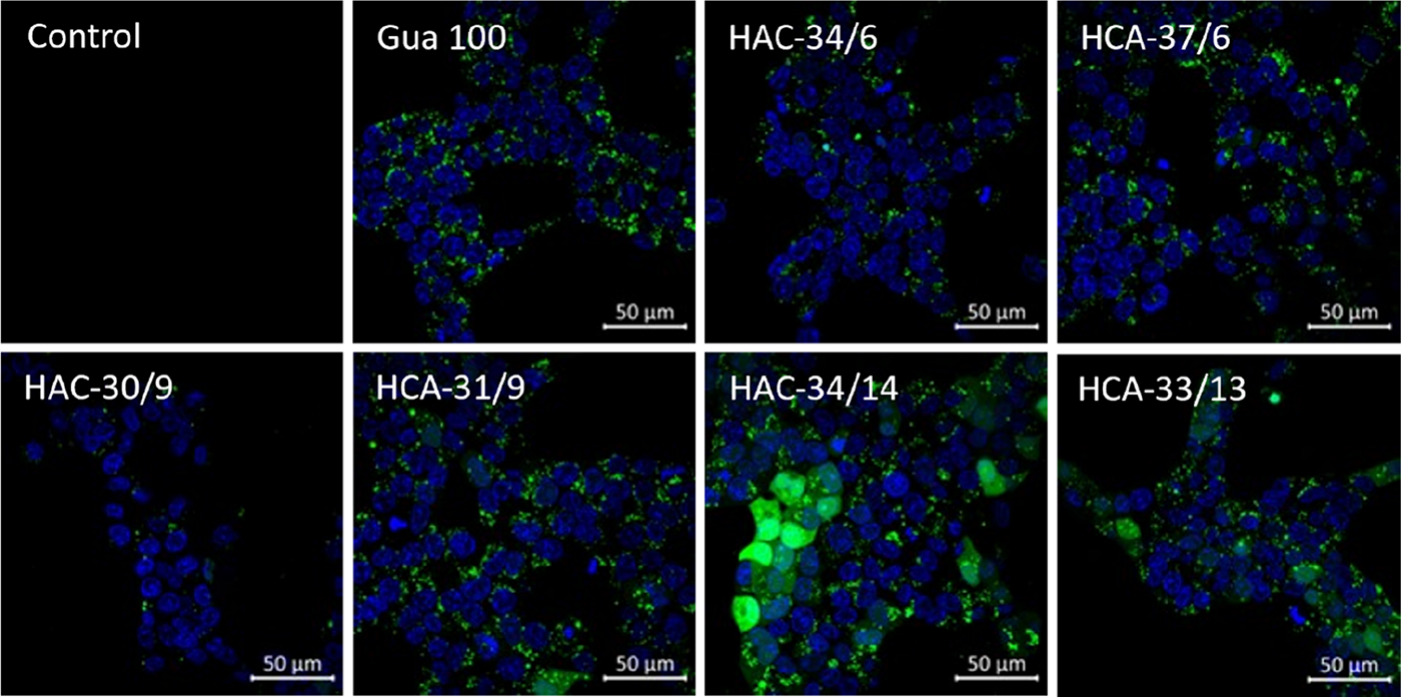
Endosomal release was analyzed via confocal laser scanning microscopy (CLSM) following simultaneous incubation with the non-permeable dye calcein with a final concentration of 25 μg mL^–1^ (green) and micelleplexes with N*/P 20 with a pDNA concentration of 3 μg mL^–1^ on HEK293T cells over 6 h incubation in D10H and following incubation in D20 (6 + 2 h). The cell nuclei were stained with Hoechst 33342 (blue). Green dots indicate endocytotic uptake of calcein within cellular compartments, and the diffuse green fluorescence pattern indicates calcein released to the cytosol. Non-treated and non-stained cells were used as the control.

In the case of the more cytocompatible micelles with increased content of CEAm (**HCA**-30/9 and **HCA**-33/13), the **HCA** micelles demonstrated superior transfection efficiencies in D2H in comparison to the **HAC** micelles. The order of the blocks therefore was a decisive factor and an outer anionic block appeared to be beneficial for improved transfection efficiencies, while maintaining good cytocompatibility. For the micelles with only 6 mol % CEAm (**HAC**-34/6 and **HCA**-37/6) slightly higher transfection efficiencies were achieved for the **HAC** micelles compared to its counterpart (**HCA** micelle) in D2H. In this case, the CEAm content was probably too low to result in performance differences for different block arrangements.

The transfection efficiencies in D10H were similar for both **HAC**-34/6 and **HCA**-37/6 and no significant differences in efficiency were observed between D2H and D10H (Figure 4B: plot above). Interestingly, **HAC**-34/14 and **HCA**-33/13 demonstrated the highest transfection efficiency in D10H among all tested materials despite the highest anionic CEAm content in the outer shell (**HCA**-33/13), which is usually considered to reduce the efficacy of a system. This is in contrast to common design principles using the cationic block at the outside due to the enhanced accessability of cationic charges for the genetic material. As this result was not expected, the membrane interaction was investigated in more detail, where commonly cationic groups play a crucial role. These are presumably less present on the outside of **HCA** micelles (e.g., **HCA**-33/13), but micelles are known to be a dynamic system and interaction with the middle block cannot be excluded. A modified hemolysis assay demonstrated an alleviating hemolytic effect with increasing molar content of anionic moiety (Figure S22A). This could be attributed to an increasing charge compensation of the excess of cationic groups, which reduced the interaction of the micelles with the membrane at physiological conditions. If the pH value decreases, as for example during endosomal uptake, the carboxylic groups become protonated and the compensation is diminished, which was also exemplified in an enhanced erythrocyte aggregation rate at pH 6 compared to pH 7.4 (Figure S22). This effect was particularly prominent for micelles with high anionic CEAm content (**HAC**-34/14 and **HCA**-33/13). Overall, the best performer **HCA**-33/13 featured an optimal membrane interaction adapting to pH changes and high cytocompatibility, which together yielded high performances in transfection.

To study the uptake and endosomal release property of the library, the membrane-impermeable dye calcein was used. The endocytotic uptake of the particles leads to the concurrent internalization of calcein (punctuate fluorescence pattern), and the endosomal release leads to release of calcein (broad cytosolic fluorescence pattern). In full growth medium (D10H), all polymers revealed a fast uptake after 6 h incubation in comparison to the non-complexed pDNA (Figures S19−S20, SI). A broad cytosolic fluorescence pattern of several cells could be observed by the micelleplexes with the highest anionic CEAm content (**HAC**-34/14 and **HCA**-33/13). A further 2 h incubation (total incubation time of 8 h, 6 + 2 h) led to increased endosomal release with the following intensity pattern: **HAC**-34/14 > **HCA**-33/13 > **HCA**-31/9 and **HCA**-37/6. The result revealed an improved endosomal release by an increase of the CEAm content and underlined the impact of the anionic CEAm block. However, the **HAC** micelle showed more intensive calcein release in comparison to the **HCA** composition (**HAC** 34/14 vs **HCA**-33/13), which both outperform micelles with lower CEAm content or the Gua 100 control (Figure 5).

Hydrophobic (*n*BA), anionic (CEAm) and cationic (GPAm) functionalized acrylate and acrylamide monomers were used to successfully synthesize triblock copolymers with narrow molar masses (*Đ* < 1.30) by RAFT polymerization. The arrangement of the segments was varied to assemble triblock micelles with a hydrophobic core and an ionic shell, which contained either (i) a middle anionic block followed by a cationic block (**HAC**) or (ii) a middle cationic segment followed by an anionic segment (**HCA**). In contrast to common polymeric gene delivery vehicels, negatively charged carboxy groups (CEAm) were incorporated as the pH-responsive functionalities while the guanidinium groups (GPAm) functioned as the positively charged functionalities irrespective of pH. The **HAC** and **HCA** polymer formed stable micelles with sizes ranging from 25 to 36 nm, which formed stable micelleplexes after complexation with pDNA with sizes < 80 nm. In general, the incorporation of anionic charged CEAm block improved endosomal release property and the integration of the anionic block in the outer shell of the **HCA** micelles increased the transfection efficiency in full growth medium with 10% serum compared to **HAC** micelles, when more than 6 mol % CEAm are incorporated in the outer shell. In addition, the cytocompatibility of the triblock micelles improved with increasing CEAm content. Our results demonstrated that the incorporation of an anionic block in the polymer triblock structure can provide an interesting alternative for using stealth moieties without reducing the gene delivery potential of the polymers. In addition, it became evident that the arrangement of the anionic block in the triblock copolymer affects hemolysis, membrane interaction, and transfection efficiency of the delivery vehicle. More detailed studies supported an unusual endosomal release mechanism due to the pH dependence of the anionic and not cationic functionality. This paves the way to novel concepts including anionic polymers for the delivery of genetic material.

## Supporting Information Available

Materials, methods, monomer and polymer syntheses, in detail characterization by ^1^H NMR, SEC, and DLS, micelle(plexe) formation, evaluation of erythrocyte aggregation, and hemolysis (PDF)
