# Supplementary material for: Tailoring Gene Transfer Efficacy through the Arrangement of Cationic and Anionic Blocks in Triblock Copolymer Micelles
Source: ACS Macro Lett. 2024 Jan 17;13(2):158–65. doi: 10.1021/acsmacrolett.3c00633 (PMC10883036; doi:10.1021/acsmacrolett.3c00633)
Supplement: Supplementary file 1 — mz3c00633_si_001.pdf [file mz3c00633_si_001.pdf]

Supporting Information

Tailoring Gene Transfer Efficacy Through the Arrangement of Cationic and Anionic Blocks in Triblock Copolymer Micelles

Katharina Leer,[a] Liên S. Reichel,[a] Mara Wilhelmi,[a] Johannes C. Brendel,[a,b] Anja Traeger[a,b]*

[a]Laboratory of Organic and Macromolecular Chemistry, Friedrich Schiller University Jena, Humboldtstrasse 10, 07743 Jena, Germany.

[b]Jena Center for Soft Matter, Friedrich Schiller University Jena, Philosophenweg 7, 07743 Jena, Germany.

*Correspondence to A. Traeger (anja.traeger@uni-jena.de)

1. Materials and methods

1.1 Materials

All chemicals were used as received unless stated otherwise. The chain transfer agent 2-(butylthiocarbonothioylthio) propanoic acid (PABTC) was prepared following a previously reported procedure.[1] Acryloyl chloride, 4-Acryloylmorpholine (NAM), 1,3,5-trioxane, 1,3-diaminopropane, 1,4-dioxane (anhydrous, 99.8%) and sodium acetate trihydrate (NaOAc×3H_2O) were obtained from Sigma-Aldrich. Trifluoroacetic acid (TFA) and *N'*-bis(*tert*-butoxycarbonyl)-1H-pyrazol-1-carboxamine were obtained from TCI. Sodium sulfate (Na_2SO_4) was obtained from Grüssing GmbH. Acetic acid glacial (HOAc), sodium chloride (NaCl) and sodium hydrogencarbonate ($NaHCO_3$) were obtained from Fisher Chemical. Triethylamine (TEA) was obtained from Carl Roth. 4-Acryloylmorpholine (NAM) was obtained from Acros Organics. *N*,*N*-Dimethylacetamide (DMAc, reagent grade, 99%) was obtained from Honeywell International Inc. *β*-Alanine *tert*-butyl ester hydrochloride, 4 M hydrochloric acid (HCl) in 1,4-dioxane, *n*-butyl acrylate (*n*BA) and di-*tert*-butyldicarbonate were obtained from Alfa Aeasar. V-65B (2,2'-azobis(2,4-dimethylvaleronitrile)) was obtained from FUJIFILM Wako Chemicals. NAM, *n*BA, and 1,4-dioxane were stored over inhibitor remover beads (for hydroquinone and 4-methoxyphenol) at 4 °C. Dichloromethane (DCM) was obtained from VWR from a solvent purification system (SPS) on site. Tetrahydrofuran (THF), methanol (MeOH), *n*-hexane, ethyl acetate, and diethyl ether were distilled on-site.

For biological investigations all the following materials were ordered from the suppliers stated in brackets: TC treated cell culture flasks (Greiner Bio-One International GmbH and Labsolute, Th. Geyer GmbH & Co. KG), TC treated multi-well cell culture plates (VWR International GmbH), L929 cells (CLS Cell Lines Service GmbH, Eppelheim, Germany) HEK293T (DSMZ, Braunschweig, Germany), Dulbecco's modified eagle medium (DMEM) and 4-(2-hydroxyethyl)-1-piperazineethanesulfonic acid (HEPES) buffer 1M, phosphate-buffered saline (PBS), fetal bovine serum (FBS), Trypsin-EDTA and Penicillin-Streptomycin were purchased from Capricorn Scientific GmbH, Germany. PrestoBlue™ cell viability reagent (Thermo Fisher Scientific), linear poly(ethylene imine) (LPEI, 25 kDa, Polysciences), D (+)-Glucose (Carl Roth, Germany), Triton X-100 (Sigma-Aldrich, USA). Human blood was obtained from the Department of Transfusion Medicine of the University Hospital, Jena. pDNA encoding the enhanced green fluorescent protein (EGFP) for transfection studies was isolated with the Giga Plasmid Kit (Qiagen, Germany) from E. coli containing pEGFP-N1 (4.7 kb, Clontech, USA), pKMyc was a gift from Ian Macara (Addgene plasmid #19400;http://n2t.net/addgene: 19400;RRID: Addgene_19400). Addgene_19400), Hoechst (Invitrogen), Calcein and Hanks'5 balanced salt solution (Sigma Aldrich), 8 well chamber slide (ibidi GmbH).

1.2 Instruments

^{1}H NMR (300 MHz) spectra and ^{13}C NMR (75 MHz) spectra were measured on a spectrometer from Bruker equipped with an Avance I console, a dual ^{1}H and ^{13}C sample head and a 60 × BACS automatic sample changer. The signals were determined by using the residual solvent as the reference. All NMR spectra were analyzed with ACD/Spectrus Processor 2019.1.3. Chemical shifts (δ) are reported in ppm relatively to the solvent.

SEC was conducted on a DMAc-SEC using an Agilent 1200 series instrument equipped with differential refractive index (DRI) and UV/vis (DAD) detector. The liquid chromatography system used 1 × PSS GRAM 30 Å column (300 × 0.8 mm, 10 µm particle size) and 1 × PSS GRAM 1000 Å column (300 × 0.8 mm, 10 µm particle size). The DMAc eluent contained

0.21 wt.% LiCl as additive. Samples were run at 1 mL min^{-1} at 40 °C. Analyte samples were filtered through a polytetrafluoroethylene (PTFE) membrane with 0.45 µm pore size prior to injection. Poly(methyl methacrylate) (PMMA) narrow standards were used to calibrate the SEC system. Experimental $M_{n,SEC}$ and $Đ$ (M_w/M_n) values of synthesized polymers were determined using PSS WinGPC UniChrom GPC software. The SEC measurements in acidic solution were carried out on a Jasco system equipped with a AS-1555 Plus autosampler, a DG-980-50 degasser, a PU-980 pump, a RI-930 Plus RI detector, a Jasco oven and a PSS NOVEMA-MAX column 30/1000/1000 Å (5 µm particle size). A mixture of 0.1 M NaCl + 0.1 wt.% TFA (pH < 2) was used as an eluent at a flow rate of 1 mL min^{-1} and an oven temperature of 30 °C. Analyte samples were filtered through a polyamide membrane with 0.45 µm pore size prior to injection. Poly(2-vinylpyridine) standards (620-1,160,000 g mol^{-1}) were used to calibrate the system.

Dynamic light scattering (DLS) and Electrophoretic Light Scattering (ELS) was performed on a Zetasizer Nano ZS (Malvern Instruments, Herrenberg, Germany). All measurements were performed in disposable folded capillary cells (DTS1070, Malvern Instruments, Herrenberg, Germany). Each sample was measured in triplicates at 25 °C with measurement duration of five times 60 s after an equilibration time of 60 s and automatic attenuation selection (λ_{Ex} = 633 nm). Scattered light was detected at an angle of 173°. The intensity-weighted mean hydrodynamic sizes (Z-Average value) are based on the Stokes–Einstein relation and the corresponding polydispersity index (PDI) of the samples originated from the cumulants method. Subsequently, ζ-potential of the samples was measured in triplicates at 25 °C and 40 mV with measurement duration set to automatic (10-20 runs) after an equilibration time of 30 s and with a delay of 30 s between each measurement.

Flow cytometry. Flow cytometry was conducted on CytoFLEX Beckmann Coulter, Brea, CA, U.S. For each experiment, 204 cells per sample were analyzed using using bandpass filter 525/40 nm (FITC channel).

Multi-plate reader. PrestoBlue™ assay and hemolysis assay were measured with the multi-plate reader Tecan infinite M200Pro, Germany, using settings described in the respective method section.

Cell counter. fluidlab R-300 anvajo GmbH, Dresden, Germany.

1.3 Synthesis of monomers

Synthesis of 2-(tert-butoxycarbonyl)ethyl acrylamide (CEAm^{tB}) was performed according to a procedure from literature.[2] *β*-Alanine *tert*-butyl ester hydrochloride (10.05 g; 55.32 mmol) was dissolved in 100 mL chloroform in a 250 mL two-neck-round-bottomed-flask. The solution was purged with argon for 10 min, cooled in an ice bath and TEA was added (9.30 mL, 66.72 mmol). Subsequently, acryloyl chloride (4.50 mL, 55.35 mmol) dissolved in 50 mL chloroform in a pressure-equalizing dropping funnel was added dropwise over 1 h to the cooled solution. The solution was stirred at room temperature overnight. The resulting clear solution was washed with saturated $NaHCO_3$ (3 × 100 mL) and brine (3 × 100 mL). The aqueous layer was extracted with DCM (2 × 50 mL), the organic layers were combined, dried over Na_2SO_4 and the solvent was removed under reduced pressure. The crude product was dissolved in 200 mL diethyl ether and stored overnight in the freezer (-80 °C). The resulting suspension was filtered and the precipitate was washed with diethyl ether (1 × 50 mL) to obtain a white solid, which was dried *in vacuo* (5.67 g, 28.46 mmol, 51%; for ^{1}H and ^{13}C NMR see **Figure S2**).

^{1}H NMR (300 MHz, $CDCl_3$): δ = 1.45 (s, 9 H; −O−C(C***H*$_3$**)), 2.48 (t, ^3J = 6.0 Hz, 2 H; −C***H*$_2$**−(C=O)O−), 3.56 (q, ^3J(H,H) = 6.0 Hz, 2 H; −C***H*$_2$**−NH(C=O)−), 5.63 (dd, ^3J(H,H) = 10 Hz, ^2J(H,H) = 1.5 Hz, 1 H; −(C=O)CH=C***H*$_2$**), 6.08 (dd, ^3J(H,H) = 18 Hz, ^3J(H,H) = 10 Hz, 1 H; −(C=O)CH=C***H*$_2$**), 6.23-6.30 (m, 2 H; −(C=O)−C***H***=CH$_2$, −CH$_2$−N***H***(C=O)−) ppm.

^{13}C NMR (75 MHz, $CDCl_3$): δ = 28.1 (−O−C(***C***H$_3$)$_3$), 35.0 (−***C***H$_2$−CH$_2$−, −CH$_2$−***C***H$_2$−), 81.2 ((−O−***C***(CH$_3$)$_3$), 126.3 (***C***H$_2$=CH−), 130.9 (CH$_2$=***C***H−(C=O)−), 165.3 (CH$_2$=CH−(***C***=O)−), 172.1 (−CH$_2$−(***C***=O)−O−) ppm.

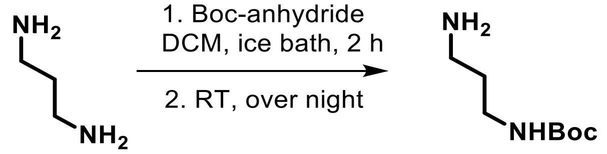

1,3-diaminopropane (1)

Synthesis of N-tert-butoxycarbonyl-1,2-diaminopropane **(1)** was performed according to an adapted procedure from literature.[3] *1,3*-Diaminopropane (22.50 mL; 269.53 mmol) was dissolved in 365 mL DCM in a 500 mL two-neck-round-bottomed-flask and cooled in an ice bath. Subsequently, di-*tert*-butyl dicarbonate (11.81 g, 54.11 mmol) dissolved in 40 mL DCM in a pressure-equalizing dropping funnel was added dropwise over 2 h to the cooled solution. The solution was stirred at room temperature overnight. DCM was removed under reduced pressure, the crude product was dissolved in destilled water and the precipitate was filtered off. The aqueous solution was extracted with ethylacetate (3 × 200 mL). The organic layer was washed with saturated $NaHCO_3$ (3 × 100 mL) and brine (3 × 100 mL). The organic layer was dried over Na_2SO_4 and the solvent was removed under reduced pressure to obtain a white solid, which was dried *in vacuo* (3.05 g, 17.50 mmol, 32%; for ^{1}H and ^{13}C NMR see **Figure S3**).

^{1}H NMR (300 MHz, $CDCl_3$): δ = 1.39-1.44 (m, 9 H; −O−C(C***H*$_3$**)$_3$), 1.62 (quin, ^3J(H,H) = 6.0 Hz, 2 H; −CH$_2$−C***H*$_2$**−CH$_2$−), 2.77 (t, ^3J(H,H) = 6.0 Hz, 2 H; −CH$_2$−CH$_2$−C***H*$_2$**−NH$_2$) 3.14-3.25 (m, 2 H; −C***H*$_2$**−NH(C=O)−), 4.90 (b, 1 H; −CH$_2$−N***H***(C=O)−) ppm.

^{13}C NMR (75 MHz, $CDCl_3$): δ = 28.4 (−O−C(***C***H$_3$)$_3$), 33.3 (−CH$_2$−***C***H$_2$−CH$_2$−), 38.4 (−***C***H$_2$−NH(C=O)−), 39.7 (−CH$_2$−CH$_2$−***C***H$_2$−NH$_2$), 79.1 (−O−***C***(CH$_3$)$_3$), 156.1 (−CH$_2$−NH(***C***=O)−) ppm.

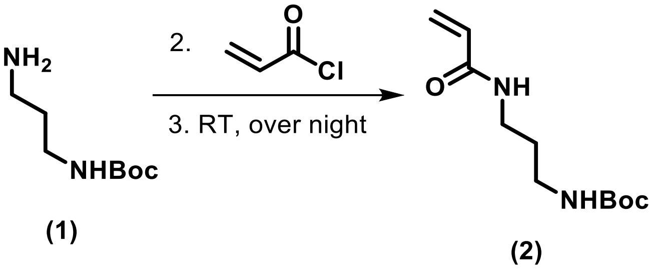

(1) (2)

Synthesis of N-tert-butoxycarbonyl-N‘-aryloyl-1,2-diaminopropane **(2)** was performed according to an adapted procedure from literature.[3] **(1)** (3.05 g, 17.50 mmol) was dissolved in 30 mL dry DCM in a 250 mL two-neck-round-bottomed-flask equipped with a magnetic stirring bar and pressure-equalizing dropping funnel, cooled in an ice bath and purged with argon for 30 min. Then, triethylamine (3.00 mL, 21.52 mmol) was added. Acryloyl chloride (1.80 mL, 22.15 mmol) dissolved in 50 mL dry DCM was added over 1.5 h. The reaction

mixture was stirred overnight at room temperature. The solution was washed with saturated $NaHCO_3$ (3 × 50 mL) and brine (2 × 50 mL). The aqueous layer was extracted with DCM (2 × 100 mL). The organic layers were combined and dried over Na_2SO_4 and the solvent was removed under reduced pressure. The obtained white solid was purified *via* flash chromatography (silica 80 g, hexane/ethyl acetate 1/1 with a gradient to more ethyl acetate, $R_f = 0.10$) to yield a white solid (2.41 g, 10.56 mmol, 60%; for ^1H and ^{13}C NMR see **Figure S4**).

^{1}H NMR (300 MHz, $CDCl_3$): δ = 1.44 (s, 9 H; $-O-C(C\boldsymbol{H}_3)_3$), 1.65 (quin, ^3J(H,H) = 6.0 Hz, 2 H; $-CH_2-C\boldsymbol{H}_2-CH_2-$), 3.18 (q, ^3J(H,H) = 6.0 Hz, 2 H; $-C\boldsymbol{H}_2-NH-(C{=}O)O-$), 3.38 (q, ^3J(H,H) = 6.0 Hz, 2 H; $-C\boldsymbol{H}_2-NH-(C{=}O)NH-$), 4.97 (b, 1H; $-CH_2-N\boldsymbol{H}-(C{=}O)O-$), 5.63 (dd, ^3J(H,H) = 10.0 Hz, ^2J(H,H) = 1.5 Hz, 1 H; $-(C{=}O)CH{=}C\boldsymbol{H}_2$), 6.13 (dd, ^3J(H,H) = 17.0 Hz, ^3J(H,H) = 10.0 Hz, 2 H; $-(C{=}O)C\boldsymbol{H}{=}CH_2$), 6.28 (dd, ^3J(H,H) = 17.0 Hz, ^2J(H,H) = 1.5 Hz, 1 H; $-(C{=}O)CH{=}C\boldsymbol{H}_2$), 6.55 (br, 1 H; $-CH_2-N\boldsymbol{H}-(C{=}O)CH{=}CH_2$) ppm.

^{13}C NMR (75 MHz, $CDCl_3$): δ = 28.4 ($-O-C(\boldsymbol{C}H_3)_3$), 30.2 ($-CH_2-\boldsymbol{C}H_2-CH_2-$), 35.8 ($-\boldsymbol{C}H_2-NH-(C{=}O)CH{=}CH_2$), 37.0 ($-\boldsymbol{C}H_2-NH-(C{=}O)O-$), 79.4 ($-O-\boldsymbol{C}(CH_3)_3$), 126.1 ($\boldsymbol{C}H_2{=}CH-$), 131.1 ($CH_2{=}\boldsymbol{C}H-(C{=}O)NH-$), 156.8 ($-NH-(\boldsymbol{C}{=}O)O-$), 165.9 ($CH_2{=}CH-(\boldsymbol{C}{=}O)NH-$) ppm.

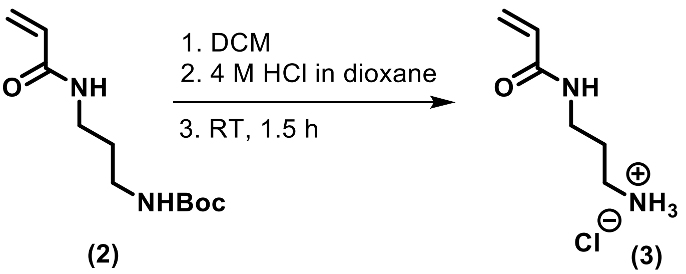

(2) (3)

Synthesis of N-Acryloyl-1,3-diaminopropane hydrochloride **(3)** was performed according to an adapted procedure from literature.[4] **(2)** (3.55 g, 15.55 mmol) was dissolved in 17 mL DCM in a 100 mL round-bottomed-flask equipped with a magnetic stirring bar and septum, cooled in an ice bath and purged with argon for 30 min. Then, 20 mL of a 4 M HCl solution in dioxane was added dropwise with a syringe. The reaction mixture was stirred for 1.5 h at room temperature and the solvent was blown off overnight using compressed air. The crude product was triturated with diethyl ether and dried *in vacuo* (2.03 g, 12.33 mmol, 79%; for ^1H and ^{13}C NMR see **Figure S5**).

^{1}H NMR (300 MHz, D_2O): δ = 1.69 (quin, ^3J(H,H) = 7.0 Hz, 2 H; $-CH_2-C\boldsymbol{H}_2-CH_2-$), 3.00 (t, ^3J(H,H) = 7.0 Hz, 2 H; $-CH_2-C\boldsymbol{H}_2-NH_3^+$) 3.34 (t, ^3J(H,H) = 7.0 Hz, 2 H; $-C\boldsymbol{H}_2-NH(C{=}O)-$), 5,74 (dd, ^3J(H,H) = 9.4 Hz, ^3J(H,H) = 2.1 Hz, 1 H; $-(C{=}O)CH{=}C\boldsymbol{H}_2$), 6.12-6.29 (m, 2 H; $-(C{=}O)CH{=}C\boldsymbol{H}_2$, $-(C{=}O)C\boldsymbol{H}{=}CH_2$) ppm.

^{13}C NMR (75 MHz, D_2O): δ = 29.2 ($-CH_2-\boldsymbol{C}H_2-CH_2-$), 38.7 ($-\boldsymbol{C}H_2-NH(C{=}O)-$), 39.6 ($-CH_2-\boldsymbol{C}H_2-NH_3^+$), 130.1 ($\boldsymbol{C}H_2{=}CH-$), 132.2 $CH_2{=}\boldsymbol{C}H-$), 171.5 ($-CH_2-NH(\boldsymbol{C}{=}O)-$) ppm.

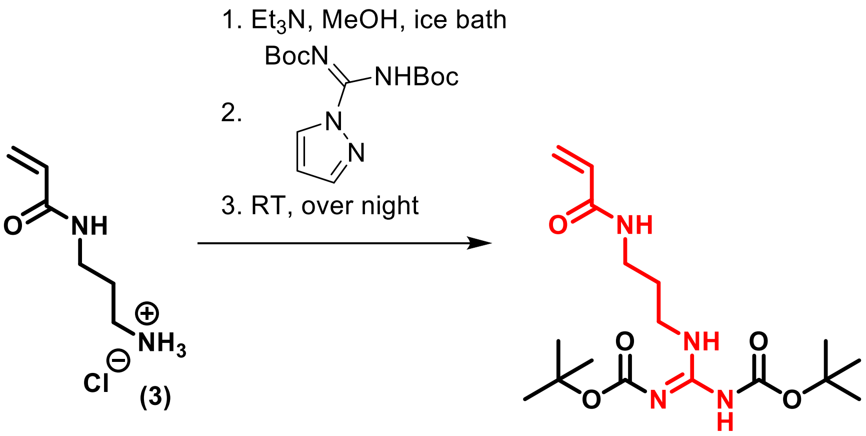

GPAm^{diBoc}

Synthesis of 1,3-di-boc-guanidinopropyl acrylamide (GPAm^{diBoc}) was performed according to an adapted procedure from literature.[4] **(3)** (2.03 g, 12.33 mmol) was dissolved in 50 mL MeOH in a 250 mL two-neck-round-bottomed-flask equipped with a magnetic stirring bar and pressure-equalizing dropping funnel, cooled in an ice bath and purged with argon for 30 min. Then, triethylamine (10.50 mL, 85.38 mmol) was added. *N*,*N'*-Di-boc-1*H*-pyrazol-1-carbamidin (4.73 g, 15.24 mmol) dissolved in 50 mL MeOH was added dropwise over 1 h. The reaction mixture was stirred overnight at room temperature. The solvent was removed under reduced pressure and the crude product was dissolved in 100 mL DCM, washed with saturated $NaHCO_3$ (3 × 50 mL) and brine (2 × 50 mL). The aqueous layer was extracted with DCM (2 × 50 mL), the organic layers were combined and dried over Na_2SO_4 and the solvent was removed under reduced pressure. The obtained yellow oil was purified *via* flash chromatography (silica, hexane/ethyl acetate 70/30 with a gradient to more ethyl acetate, R_f = 0.25) to obtain a white solid (4.14 g, 11.18 mmol, 91%; for ^{1}H and ^{13}C NMR see **Figure S6**).

^{1}H NMR (300 MHz, $CDCl_3$): δ = 1.49 (s, 18 H; −O−C(C***H***$_3$)$_3$), 1.61-1.72 (m, 2 H; −CH$_2$−C***H***$_2$−CH$_2$−), 3.33 (q, ^3J(H,H) = 6.0 Hz, 2 H; −C***H***$_2$−NH−(C=O)−), 3.49 (q, ^3J(H,H) = 6.0 Hz, 2 H; −C***H***$_2$−NH−(C=N−)NH−), 5.55 (dd, ^3J(H,H) = 7.1 Hz, ^2J(H,H) = 4.8 Hz 1 H; −(C=O)CH=C***H***$_2$), 6.27-6.30 (m, 2 H; −(C=O)CH=C***H***$_2$, −(C=O)C***H***=CH$_2$), 7.88 (br, 1 H; −CH$_2$−N***H***−(C=O)−), 8.46 (br, 1 H; −CH$_2$−N***H***−(C=N−)NH−), 11.44 (s, 1 H; −NH−(C=N−)−N***H***−(C=O)−O−) ppm.

^{13}C NMR (75 MHz, $CDCl_3$): δ = 28.0 (−O−C(***C***H$_3$)$_3$), 28.2 (−O−C(***C***H$_3$)$_3$), 29.7 (−CH$_2$−***C***H$_2$−CH$_2$−), 34.7 (−***C***H$_2$−NH−(C=O)−), 37.0 (−***C***H$_2$−NH−(C=N−)NH−), 79.5 (−O−***C***(CH$_3$)$_3$), 83.5 (−O−***C***(CH$_3$)$_3$), 125.3 (***C***H$_2$=CH−), 131.8 (CH$_2$=***C***H−(C=O)−), 153.1 (−NH−(***C***=O)−O−), 157.2 (−NH−(***C***=N−)−NH−), 162.9 (−NH−(***C***=O)−O−), 165.5 (CH$_2$=CH−(***C***=O)−) ppm.

1.4 Synthesis of polymers and micelle assembly

Calculations for RAFT polymerization. The conversion *p* of each monomer was analyzed using ^{1}H NMR spectroscopy in $CDCl_3$ by comparing the integrals of the vinyl peaks of each monomer against an internal reference (1,3,5-trioxane) before (t = 0) and after (t = final) polymerization. For *n*BA, the conversion was investigated by comparing the integral of the signal at 5.79 ppm, belonging to one of the vinyl protons of *n*BA, against the integral of the signal at 5.08-5.12 ppm, assigned to the internal NMR reference 1,3,5-trioxane. The conversion of GPAm^{diBoc} and NAM were determined by comparing the integrals of the signals at 5.44-5.54 ppm, belonging to one of the vinyl protons of GPAm^{diBoc}, and at 5.59-5.67 ppm, attributed to one of the vinyl protons

of NAM, against the integral of the signal at 5.02-5.11 ppm, assigned to the internal reference 1,3,5-trioxane. The conversion of CEAm^{tB} and NAM were studied by comparing the integrals of the signals at 5.42-5.50 ppm, belonging to one of the vinyl protons of CEAm^{tB}, and at 5.53-5.64 ppm, attributed to one of the vinyl protons of NAM, against the integral of the signal at 4.96-5.07 ppm, assigned to the internal reference 1,3,5-trioxane.

The theoretical degree of polymerization (DP_{th}) was calculated by multiplying the conversion p with the targeted DP for each monomer (**Equation S1**).

$$DP_{\mathrm{th}} = \frac{[M]_0}{[CTA]_0} \cdot p = DP_{\mathrm{targeted}} \cdot p \qquad \textbf{(S1)}$$

The theoretical number-average molar mass ($M_{\mathrm{n,th}}$) was calculated with **Equation S2**:

$$M_{\mathrm{n,th}} = \frac{[M]_0 p M_{\mathrm{M}}}{[CTA]_0} + M_{\mathrm{CTA}} \qquad \textbf{(S2)}$$

$[M]_0$ and $[CTA]_0$ are the initial concentrations of monomer and chain transfer agent (CTA), respectively. p is the monomer conversion. M_{M} and M_{CTA} are the molar masses of the monomer and CTA, respectively.

Procedure for the synthesis of Gua 100 (P(GPAm)$_{71}$). The polymerization was performed as described before.[2] PABTC (86.50 mg of a 5 wt% solution in DMAc), GPAm^{diBoc} (670.00 mg, 1.80 mmol), 1,4-dioxane (854.60 mg), DMAc (309.60 mg), V-65B (147.6 mg of a 1 wt% solution in 1,4-dioxane) and 1,3,5-trioxane (13.20 mg) as an internal NMR reference were introduced to a vial equipped, which was sealed with a cap. The mixture was deoxygenated by bubbling argon through the solution for 10 min. The vial was then transferred to a preheated thermostated oil bath set at 45 °C. After a polymerization time of 5 h, the vial was cooled to room temperature and exposed to air. 2-3 droplets of the polymerization mixture were used for ^{1}H NMR spectroscopy and SEC analysis. The crude polymer was dissolved in 10 mL THF and precipitated in -80 °C hexane (3 × 40 mL). Finally, the polymer was dried under vacuum. The polymer was introduced to a 25 mL round-bottom flask and TFA/deionized water (97/3, v/v%) was added to reach a concentration of 100 mg mL^{-1}. The solution was stirred for 3 h at RT and the TFA was blown off overnight using compressed air. Subsequently, the crude deprotected polymer was precipitated three times from MeOH (10 mL) into -80 °C cold diethylether (3 × 40 mL). Finally, the deprotected polymer was dried under vacuum and analzed by ^{1}H NMR spectroscopy and SEC. (**Figure S7**).

General procedure for the synthesis of P(nBA). The polymerization was performed as described before.[2] PABTC, *n*BA , 1,4-dioxane , V-65B (1 wt% solution in 1,4-dioxane) and 1,3,5-trioxane as an internal NMR reference (**Table S1**) were introduced to a 50 mL round-bottomed-flask equipped with a magnetic stirring bar which was sealed with a rubber septum. The mixture was degassed by bubbling argon through the solution for 20 min. The vial was then transferred to a preheated thermostated oil bath set at 50 °C. After a polymerization time of 4 h, the flask was cooled to room temperature and exposed to air. 2-3 droplets of the polymerization mixture were used for ^{1}H NMR and SEC analysis. Afterward, the solvent was removed under reduced pressure, the crude polymer was dissolved in 15 mL THF and precipitated twice into a mixture of MeOH/H_2O (80/20). Finally, the polymer was dried under vacuum and analzed by ^{1}H NMR spectroscopy and SEC (**Figure S8, Table S6 and Figure S12**).

General procedure for the synthesis of P(nBA)-b-P(CEAm^{tB}-co-NAM). The polymerization was performed as described before.[2] P(*n*BA), CEAm^{tB}, NAM, 1,4-dioxane, DMAc, V-65B (1 wt% solution in 1,4-dioxane) and 1,3,5-trioxane as an internal NMR

reference (**Table S2**) were introduced to a vial equipped with a magnetic stirring bar which was sealed with a cap. The mixture was degassed by bubbling argon through the solution for 10 min. The vial was then transferred to a preheated thermostated oil bath set at 45 °C. After the targeted conversion was achieved, the vial was cooled to room temperature and exposed to air. The polymerization mixture was then dissolved in 7 mL THF and precipitated in -80 °C hexane (3 × 30 mL). Finally, the polymer was dried under vacuum and analzed by ^{1}H NMR spectroscopy and SEC (**Figure S8, Table S6 and Figure S13**).

General procedure for the synthesis of P(nBA)-b-P(GPAm^{diBoc}). The polymerization was performed as described before.[2] P(*n*BA), GPAm^{diBoc}, 1,4-dioxane, DMAc, V-65B (1 wt% solution in 1,4-dioxane) and 1,3,5-trioxane as an internal NMR reference (**Table S3)** were introduced to a vial equipped with a magnetic stirring bar which was sealed with a cap. The mixture was degassed by bubbling argon through the solution for 10 min. The vial was then transferred to a preheated thermostated oil bath set at 45 °C. After the targeted conversion was achieved, the vial was cooled to room temperature and exposed to air. The polymerization mixture was then dissolved in 7 mL THF and precipitated in -80 °C hexane (3 × 30 mL). Finally, the polymer was dried under vacuum and analzed by ^{1}H NMR spectroscopy and SEC (**Figure S8, Table S6 and Figure S14**).

General procedure for the synthesis of P(nBA)-b-P(CEAm^{tB}-co-NAM)-P(GPAm^{diBoc}) (HAC^{pro}). BC^{tB}N, GPAm^{diBoc}, 1,4-dioxane, DMAc, V-65B (1 wt% solution in 1,4-dioxane) and 1,3,5-trioxane as an internal NMR reference (**Table S4**) were introduced to a vial equipped with a magnetic stirring bar which was sealed with a cap. The mixture was degassed by bubbling argon through the solution for 10 min. The vial was then transferred to a preheated thermostated oil bath set at 45 °C. After the targeted conversion was achieved, the vial was cooled to room temperature and exposed to air. The polymerization mixture was then dissolved in 7 mL THF and precipitated in -80 °C hexane (3 × 30 mL). Finally, the polymer was dried under vacuum and analzed by ^{1}H NMR spectroscopy and SEC (**Figure S8 and S13**).

General procedure for the synthesis of P(nBA)-b-P(GPAm^{diBoc})-b-P(CEAm^{tB}-co-NAM) (HCA^{pro}). BG^{diBoc}, CEAm^{tB}, NAM, 1,4-dioxane, DMAc, V-65B (1 wt% solution in 1,4-dioxane) and 1,3,5-trioxane as an internal NMR reference (**Table S5**) were introduced to a vial equipped with a magnetic stirring bar which was sealed with a cap. The mixture was degassed by bubbling argon through the solution for 10 min. The vial was then transferred to a preheated thermostated oil bath set at 45 °C. After the targeted conversion was achieved, the vial was cooled to room temperature and exposed to air. The polymerization mixture was then dissolved in 7 mL THF and precipitated in -80 °C hexane (3 × 30 mL). Finally, the polymer was dried under vacuum and analzed by ^{1}H NMR spectroscopy and SEC (**Figure S8 and S14**).

Table S1. Amount of different substances used for the polymerization of the macroCTA P(nBA)$_{78}$ and two batches of P(nBA)$_{80}$.

Polymer	P(nBA)$_{78}$	P(nBA)$_{80}$ (1)	P(nBA)$_{80}$ (2)
**Monomer**	***n*BA**	***n*BA**	***n*BA**
**DP$_{targeted}$**	100	100	100
**DP$_{th}$**	**78**[b)]	**80**[b)]	**80**[b)]
**Conversion [%]**	**78.8**	**80.9**	**80.2**
**m(PABTC) [mg]**	**161.04**	**102.24**	**41.49**
n(PABTC) [mmol]	0.68	0.43	0.17
**m(Mon) [g]**	**8.672**	**5.501**	**2.230**
n(Mon) [mmol]	67.66	42.92	17.40
**m(V-65-sol.)[a] [mg]**	**750.40**	**481.80**	**201.60**
m(V-65) [mg]	7.50	4.81	2.02
n(V-65) [mmol]	2.90×10^{-2}	1.86×10^{-2}	7.82×10^{-3}
**m(dioxane) [g]**	**9.201**	**5.836**	**2.370**
[Mon]$_0$ [mol L^{-1}]	3.50	3.50	3.49
[V-65]$_0$ [mol L^{-1}]	1.50×10^{-3}	1.51×10^{-3}	1.57×10^{-3}
[CTA]$_0$ [mol L^{-1}]	3.51×10^{-2}	3.50×10^{-2}	3.41×10^{-2}
[CTA]/[V-65]	23.40	23.18	21.72
T [°C]	50	50	50
time [h]	4.0	4.0	4.0

a) Added mass of a 1 wt% solution of V-65B in 1,4-dioxane.
b) calculated using **Equation S1.**

Table S2. Summary of the conditions used for the polymerization of the second block to obtain the diblock copolymers P[(nBA)$_x$-b-P(CEAm^{tB}$_y$-co-NAM$_z$)].

Polymer	P[(nBA)$_x$-b-(CEAm^{tB}$_y$-co-NAM$_z$)]					
	**x: 78, y: 11, z: 35**		**x: 78, y: 17, z: 37**		**x: 80, y: 30, z: 30**	
**Block**	**P(nBA)$_{78}$**		**P(nBA)$_{78}$**		**P(nBA)$_{80}$ (1)**	
**Monomer**	**CEAm^{tB} c**	**NAM**	**CEAm^{tB}**	**NAM**	**CEAm^{tB}**	**NAM**
**DP$_{targeted}$**	16	40	25	42	39	33
**DP$_{th}$**	**11**[b)]	**35**[b)]	**17**[b)]	**37**[b)]	**30**[b)]	**30**[b)]
**Conversion [%]**	**71.2**	**88.6**	**71.2**	**88.6**	**77.4**	**93.1**
**m(Block 1) [mg]**	**598.10**		**601.10**		**819.80**	
n(Block 1) [mmol]	5.78×10^{-2}		5.81×10^{-2}		7.73×10^{-2}	
**m(Mon) [mg]**	**193.90**	**330.70**	**293.20**	**349.70**	**611.50**	**361.90**
n(Mon) [mmol]	0.97	2.34	1.47	2.48	3.07	2.56
**m(V-65-sol.)[a] [mg]**	**423.00**		**512.00**		**729.20**	

	P[(nBA)$_x$-b-(CEAm$^{tB}{}_y$-co-NAM$_z$)]		
m(V-65) [mg]	4.23	5.12	7.29
n(V-65) [mmol]	1.64×10^{-2}	1.98×10^{-2}	2.82×10^{-2}
**m(dioxane) [mg]**	**758.30**	**924.60**	**1775.50**
**m(DMAc) [mg}**	**714.0**	**865.9**	**953.80**
[Mon]$_0$ [mol L^{-1}]	1.51	1.51	1.49
[V-65]$_0$ [mol L^{-1}]	7.46×10^{-3}	7.55×10^{-3}	7.44×10^{-3}
[CTA]$_0$ [mol L^{-1}]	2.63×10^{-2}	2.22×10^{-2}	2.04×10^{-2}
[CTA]/[V-65]	3.53	2.94	2.74
T [°C]	45	45	45
time [h]	2.0	2.0	2.0

[a] Added mass of a 1 wt% solution of V-65B in 1,4-dioxane.
[b] calculated using **Equation S1.**

Table S3. Summary of the conditions used for the polymerization of the second block to obtain the diblock copolymers P[(nBA)$_x$-b-P(GPAm^{diBoc})$_m$].

	P[(nBA)$_x$-b-(GPAm^{diBoc})$_m$]		
**Polymer**	**x: 78, m: 61**	**x: 78, m: 73**	**x: 80, m: 69**
**Block**	**P(nBA)$_{78}$**	**P(nBA)$_{78}$**	**P(nBA)$_{80}$ (2)**
**Monomer**	**GPAm^{diBoc}**	**GPAm^{diBoc}**	**GPAm^{diBoc}**
**DP$_{targeted}$**	74	88	88
**DP$_{th}$**	**61[b)]**	**73[b)]**	**69[b)]**
**Conversion [%]**	**82.7**	**83.5**	**79.5**
**m(Block 1) [mg]**	**500.60**	**500.30**	**213.20**
n(Block 1) [mmol]	4.84×10^{-2}	4.84×10^{-2}	2.03×10^{-2}
**m(Mon) [g]**	**1.334**	**1.581**	**0.661**
n(Mon) [mmol]	3.60	4.27	1.78
**m(V-65-sol.)[a] [mg]**	**463.20**	**556.50**	**230.80**
m(V-65) [mg]	4.63	5.57	2.31
n(V-65) [mmol]	1.79×10^{-2}	2.16×10^{-2}	8.94×10^{-3}
**m(dioxane) [g]**	**1.021**	**1.509**	**0.640**
**m(DMAc) [g}**	**0.895**	**0.802**	**0.335**
[Mon]$_0$ [mol L^{-1}]	1.51	1.50	1.48
[V-65]$_0$ [mol L^{-1}]	7.50×10^{-3}	7.57×10^{-3}	7.46×10^{-3}
[CTA]$_0$ [mol L^{-1}]	2.03×10^{-2}	1.70×10^{-2}	1.69×10^{-2}
[CTA]/[V-65]	2.71	2.25	2.27
T [°C]	45	45	45

time [h]	2.5	2.5	2.5

a) Added mass of a 1 wt% solution of V-65B in 1,4-dioxane.
b) calculated using **Equation S1.**

Table S4. Summary of the conditions used for the polymerization of the third block to obtain the triblock copolymers **HAC-30/9**, **-34/6 and -34/14**.

Polymer	HAC^{pro}-30/9	HAC^{pro}-34/6	HAC^{pro}-34/14
**Block**	P[(*n*BA)$_{78}$-*b*-(CEAm^{tB}$_{17}$-*co*-NAM$_{37}$)]	P[(*n*BA)$_{78}$-*b*-(CEAm^{tB}$_{11}$-*co*-NAM$_{35}$)]	P[(*n*BA)$_{80}$-*b*-(CEAm^{tB}$_{30}$-*co*-NAM$_{30}$)]
**Monomer**	**GPAm^{diBoc}**	**GPAm^{diBoc}**	**GPAm^{diBoc}**
**DP$_{targeted}$**	68	81	88
**DP$_{th}$**	**56$^{b)}$**	**65$^{b)}$**	**72$^{b)}$**
**Conversion [%]**	**82.7**	**80.3**	**82.1**
**m(Block 2) [mg]**	**608.30**	**496.70**	**302.1**
n(Block 2) [mmol]	3.18 × 10^{-2}	2.82 × 10^{-2}	1.44 × 10^{-2}
**m(Mon) [mg]**	**805.10**	**855.50**	**470.60**
n(Mon) [mmol]	2.17	2.31	1.27
**m(V-65-sol.)[a] [mg]**	**286.20**	**302.30**	**171.3**
m(V-65) [mg]	2.86	3.02	1.71
n(V-65) [mmol]	1.11 × 10^{-2}	1.17 × 10^{-2}	0.66× 10^{-2}
**m(dioxane) [mg]**	**616.20**	**658.10**	**451.3**
**m(DMAc) [mg}**	**546.30**	**579.80**	**346.8**
[Mon]$_0$ [mol L^{-1}]	1.49	1.49	1.31
[V-65]$_0$ [mol L^{-1}]	7.63 × 10^{-3}	7.56 × 10^{-3}	6.79 × 10^{-3}
[CTA]$_0$ [mol L^{-1}]	2.19 × 10^{-2}	1.82 × 10^{-2}	1.48 × 10^{-2}
[CTA]/[V-65]	2.87	2.41	2.18
T [°C]	45	45	45
time [h]	2.5	2.5	2.0

a) Added mass of a 1 wt% solution of V-65B in 1,4-dioxane.
b) calculated using **Equation S1.**

Table S5. Summary of the conditions used for the polymerization of the third block to obtain the triblock copolymers **HCA-31/9**, **-37/6 and -33/13**.

Polymer	HCA^{pro}-31/9		HCA^{pro}-37/6		HCA^{pro}-33/13	
**Block**	P[(*n*BA)$_{78}$-*b*-(GPAm^{diBoc})$_{61}$]		P[(*n*BA)$_{78}$-*b*-(GPAm^{diBoc})$_{73}$]		P[(*n*BA)$_{80}$-*b*-(GPAm^{diBoc})$_{69}$]	
**Monomer**	**CEAm^{tB} c**	**NAM**	**CEAm^{tB}**	**NAM**	**CEAm^{tB}**	**NAM**
**DP$_{targeted}$**	26	45	17	41	41	37
**DP$_{th}$**	**17$^{b)}$**	**39$^{b)}$**	**11$^{b)}$**	**36$^{b)}$**	**27$^{b)}$**	**33$^{b)}$**

Conversion [%]	68.1	87.5	68.8	90.0	67.2	90.0
**m(Block 2) [mg]**	**731.7**		**861.20**		**175.83**	
n(Block 2) [mmol]	2.22×10^{-2}		2.29×10^{-2}		4.83×10^{-3}	
**m(Mon) [mg]**	**114.80**	**142.7**	**78.5**	**134.1**	**39.6**	**25.6**
n(Mon) [mmol]	0.58	1.01	0.39	0.95	0.20	0.18
**m(V-65-sol.)[a] [mg]**	**371.6**		**322.6**		**94.7**	
m(V-65) [mg]	3.72		3.23		0.95	
n(V-65) [mmol]	1.44×10^{-2}		1.25×10^{-2}		3.68×10^{-3}	
**m(dioxane) [mg]**	**900.10**		**639.40**		**180.7**	
**m(DMAc) [mg}**	**505.20**		**768.70**		**167.6**	
$[Mon]_0$ [mol L^{-1}]	0.84		0.72		0.83	
$[V\text{-}65]_0$ [mol L^{-1}]	7.60×10^{-3}		6.70×10^{-3}		8.01×10^{-3}	
$[CTA]_0$ [mol L^{-1}]	1.17×10^{-2}		1.23×10^{-2}		1.05×10^{-2}	
[CTA]/[V-65]	1.54		1.84		1.31	
T [°C]	45		45		45	
time [h]	2.5		2.5		2.5	

a) Added mass of a 1 wt% solution of V-65B in 1,4-dioxane.
b) calculated using **Equation S1.**

*Deprotection of **HAC**^{pro} and **HCA**^{pro}.* The deprotection was performed as described before.[2] A sample of *tert*-butyl- and Boc-protected polymer was introduced to a 25 mL round-bottom flask equipped with a magnetic stirring bar and TFA/deionized water (97/3, v/v%) was added to reach a concentration of 200 mg mL^{-1}. The solution was stirred for 3 h at RT and the TFA was blown off overnight using compressed air. Subsequently, the crude deprotected polymer was precipitated three times from THF (plus a bit MeOH to help solubility) into -80 °C cold hexane. Finally, the deprotected polymer (**HAC** and **HCA**) was dried under vacuum and analzed by ^{1}H NMR spectroscopy (**Figure S13 and Figure S14**).

Assembly of micelles. The triblock quarterpolymer samples (**HAC-30/9, -34/6, -34/14, HCA-31/9, -37/6, -33/13)** were dissolved in a mixture of THF/MeOH (80/20 v/v%) at a concentration of 8 mg mL^{-1}. Afterward, ultrapure water was added over 40 min with a syringe pump to the polymer solution to reach approximately 4 mg mL^{-1}. The polymer solutions were then dialyzed against a 20 mM NaOAc (pH = 5) buffer solution (MWCO = 3.5-5.0 kDa) and filtered with sterile PA (polyamide) syringe filters (pore size: 0.22 µm). The size and ζ-potential of the micelles was then determined by DLS measurements (**Figure S15-S16**).

1.5 Cell culture

For cultivation of the mouse fibroblast cell line L929 and human embryonic kidney cell line HEK293T cell line Dulbecco's modified Eagle's medium (DMEM, 1 g L^{-1} glucose) was used as growth medium. The growth medium was further supplemented with 10 % (v/v) fetal bovine serum (FBS) (D10), 100 U mL^{-1} penicillin, and 100 µg mL^{-1} streptomycin (D10). Cells were cultivated at 37 °C in a humidified 5% (v/v) CO_2 atmosphere.

One day before the PrestoBlue™ assay, L929 cell line was seeded in a 96 well-plate at a cell concentration of 0.1 x 10^6 cells mL^{-1} in a total volume 100 µL D10 per well.

For transfection efficiency studies, HEK293T was seeded in a 24 well-plate at a cell concentration of 0.2 x 10^6 cells mL^{-1} in 500 µL D10, supplemented with 10 mM HEPES (D10H) one day before the experiment to reach a cell confluency > 70%. One hour before the experiment started, the medium was changed to fresh D10H.

Fluidlab R-300 anvajo was used to count cells for cell seeding.

1.6 Determination of metabolic activity in L929 cells (PrestoBlue™ assay)

For the determination of cytotoxicity of the polymers the metabolic activity of viable L929 cells was measured with the PrestoBlue assay. The assay was performed based on ISO10993-5. 1 h before treatment the medium was changed to 90 µL fresh D10H. In triplicate, cells were treated with 10 µL diluted polymers, which were dilutes in 20 mM HEPES buffer supplemented with 5% (w/v) glucose (HBG buffer with pH 7.4). The tested polymer concentration is ranging from 13 to 400 µg mL^{-1}. LPEI was dilted in 20 mM HEPES buffer and was used as control at concentration of 10 to 100 µg mL^{-1}. Treated cells were incubated with polymer solution for 24 h. Afterwards the medium was replaced by a 10% (v/v) PrestoBlue solution in a fresh D10, prepared according to the manufacturer's instructions. Cells were further incubated at 37 °C for 45 min and the fluorescence was measured with the multi-plate reader at $\lambda_{Ex} = 570$ / $\lambda_{Em} = 610$ nm. With buffer treated control cells on the same plate were defined to 100% viability. Results are shown in **Figure 4A** and LC_{50} is shown in **Table S7**. The relative number of viable cells was calculated as in **Equation S3**:

$$\text{Rel. viability / \%} = \frac{FI_{Sample} - FI_0}{FI_{Ctl} - FI_0} \cdot 100 \qquad \textbf{(S3)}$$

Where FI_{Sample}, FI_0, and FI_{Ctrl} represent the fluorescence intensity of a given sample, medium without cells (the blank), and buffer-treated control (100 % viability), respectively.

1.7 Polyplexation and micelleplexation

For the polyplex preparation HBG buffer was used to dilutes plasmid DNA and polymers. Plasmid DNA was diluted to have a master mix in which the pDNA concentration is twice as highly than in the final polyplex solution. Polymers were diluted at double the concentration as aimed in the final N*/P ratio. The master mix was added 1:1 (v/v) to the diluted polymer solution. Immediately the mixture was vortexed for 10 s at maximum speed and incubated for 15 min at room temperature.

1.8 N*/P ratio calculations

The N/P ratio was calculated according to a previously published protocol.[5]

The N*/P ratio was defined as the ratio of the total amount of protonated amines in polymer solution in relation to the total amount of phosphates in the pDNA solution.

The volume of polymer needed to prepare polyplexes with 30 µg mL^{-1} pDNA at different N*/P ratios was calculated as described by the following **Equation S4**:

$$V_{total} \cdot P = V_{poly} \cdot N_{poly}$$

$$V_{poly} = \frac{V_{total} \cdot P}{N_{poly}}$$

$$V_{poly} = V_{total} \cdot \frac{n_{pDNA} \cdot P}{n_{poly} \cdot N}$$

$$V_{poly} = V_{total} \cdot \frac{m_{pDNA} \cdot P \cdot M_{poly}}{m_{poly} \cdot N \cdot M_{pDNA}} \quad \textbf{(S4)}$$

Where V_{total}, P, V_{poly} and N_{poly} are the total required volume, the total number of phosphates of the pDNA, the required volume of polymer and the total number of active amines of the polymer, respectively.

Characterization of triblock micelles and micelleplexes by DLS

The hydrodynamic size of assembled micelles were measured as described in the intrumental section.

Polyplexes were prepared as described in the method section of polyplexation and micelleplexation with pKMyc. The hydrodynamic size of the polyplexes were determined with DLS Zetasizer Nano ZS. For hydrodynamic size measurement 70 µL of the polyplexes was used and for ζ-potential determination 100 µL of the polyplexes was diluted in 900 µL HBG buffer. All measurements were performed as described in the intrumental section. Results were shown in **Figure S15-S18.**

1.9 Investigation of uptake and endosomal release

To study the endosomal escape, HEK293T cells were seeded at 0.2 x 10^6 cells mL^{-1} in D10H a 8 well chamber slide. The cells were preincubated for 24 h. 1 h before treating, 250 µL of old medium was changed to 225 µL new D10H. Firstly, a final concentration of 25 µg mL^{-1} calcein was added to the cells followed by the micelleplexes (N*/P 20 with a pDNA concentration of 3 µg $mL^{-1)}$. Cells were incubated for 6 h at 37 °C in a humidified 5% (v/v) CO_2 atmosphere (6 h). Afterwards, cells were washed twice with warm Hanks'5 balanced salt solution with addition of 2% serum and cells were further incubated in full growth medium with 20% FCS (D20).

Immediately the uptake and endosomal escape due to the distribution pattern of calcein in living HEK293T cells was imaged, using confocal laser scanning microscope (CLSM) LSM880, Elyra PS.1 system (Zeiss, Germany) was used. Therefore, the argon laser was applied for an excitation at 488 nm (1%) and 405 nm (0.5%). The emission filters 410-469 nm (Hoechst, with gain 680) and 490-544 nm (Calcein) with a gain of 700 was used. To avoid cross talk between the different channels, Hoechst 33342 and calcein were imaged simultaneously in different tracks. Images were acquired using the ZEN software, version 2.3 SP1 (Zeiss, Germany) using a magnification of 40x at 37 °C. Therefore, 40 × 1.4 NA plan apochromat oil objective was used.

For further time point, cells were further incubated in D20 for 2 h at 37 °C and imaged again after a total incubation time of 8 h (6+8 h).

1.10 Transfection efficiency

HEK293T cells were seeded at a density of 0.2 x 10^6 cells mL^{-1} in a 24-well plate in 10 % FBS full-growth medium supplemented with 1% HEPES (D10H). To reach a cell confluency of > 70%, cells were preincubated for 24 hours at 37 °C (5% CO_2) before treatment. 1 h before treatment, the medium was replaced with fresh 400 µL fresh 2% FBS growth medium supplemented with 1% HEPES (D2H) or fresh 10% FBS growth medium supplemented with 1% HEPES (D10H). The polyplexes were prepared as described in the method section of polyplexation and micelleplexation.

Cells were treated with 100 µL polyplex/micelleplex (1:5 dilution) at N*/P 20 and a final pDNA concentration of 3 µg mL^{-1} on cells. As positive control for the assay, LPEI was used. Cells

were cultivated over 24 h without medium change. After 24 h of incubation, the medium was changed to the fresh full-growth medium (D10), and cells were further incubated for another 24 h (24+24 h correspond to 48 h incubation in total). As negative controls, non-treated cells, cells treated with master mix, and cells treated with polyplexes/micelleplexes of pKMyc were taken. For harvesting, the supernatant was transferred to a new 24-well plate and cells were treated with 150 µL of Trypsin EDTA and incubating for 10 min at 37 °C (5% CO_2). To stop trypsinization, 500 µL respective supernatant was added and 250 µL of cell suspension was transferred to 96-well plate for following flow cytometry measurement using the FITC channel (λ = 525/40 nm). Viable, single cells with higher fluorescence intensity were gated into the negative control cells, which were treated with pKMyc-polymer complexes. The gating strategy was applied as shown in **Figure S1** below. Results are shown in **Figure 4B**.

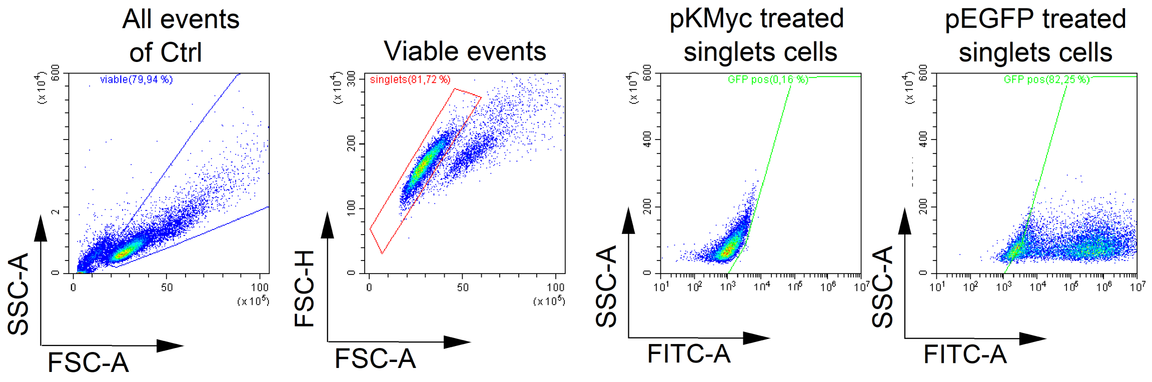

Figure S1 The gating strategy for transfection e.g., for **HCA-33/13** in D2H after 24+24 h incubation. Firstly, viable cells were gated according to FSC-A/SSC-A (blue gate) pattern of the control treated with HBG buffer. Followed by gating viable cells in FSC-H/FSC-A (red gate) to distinguish single cells to cell aggregation. Samples treated with pKMyc were gated so that EGFP positive cells in the gate was lower than 1 % (green gate). The EGFP expressing cells were gated using the gate of the pKMyc control.

1.11 Erythrocyte aggregation and hemolysis

The erythrocyte aggregation and hemolysis were performed according to a previously published protocol.[5] The release of hemoglobin from erythrocytes was examined to investigate the interaction of polymers with cellular membranes. Therefore, human blood from three different donors were collected in tubes with EDTA additive, was obtained from the Department of Transfusion Medicine of the University Hospital, Jena. To remove the serum from the erythrocytes, the blood was centrifuged without pooling at 4,500 × g for 5 min, and the supernatant (the serum) was removed. The pellet of erythrocytes was washed three times with cold phosphate-buffered saline (PBS, pH 7.4). The resulting suspension of erythrocytes was diluted 10-fold with PBS either pH 7.4 or 6.0, to imitate the cell membrane and the endosome membrane, respectively. In the next step, the polymers, which were diluted with PBS (pH = 7.4 or 6.0) to the aimed concentration, ranging from 10 µg mL^{-1} to 150 µg mL^{-1} were added to the erythrocytes. In detail, 350 µL aliquots of erythrocyte suspension were added at the ratio of 1:1 to the polymer solutions. The erythrocyte-polymer suspensions were incubated at 37 °C for 60 min and centrifugation at 2,400 × g for 5 min before the supernatant was transferred in triplicate to a clear flat bottomed 96-well plate (VWR, Germany). The hemoglobin release was determined as the hemoglobin absorption at λ = 544 nm. Absorption at λ = 630 nm was used as a reference. Complete hemolysis (100%) was achieved using 1% Triton X-100 as the positive control since Triton strongly disrupts the cell membrane. Pure PBS was used as negative control (0% hemolysis). The hemolytic activity of the polymer was calculated as follows:

$$\text{Hemolysis / \%} = \frac{(A_{Sample} - A_{Negative\ control})}{(A_{Positive\ control} - A_{Negative\ control})} \cdot 100 \quad \textbf{(S5)}$$

Where A_{Sample}, $A_{Negative\ control}$, and $A_{Positive\ control}$ are the absorption values of a given sample, the PBS treatment, and the Triton X-100 treatment, respectively. A value less than 2% hemolysis rate was classified as non-hemolytic, 2 to 5% as slightly hemolytic, and values > 5% as hemolytic.

To determine the cell aggregation, erythrocytes were isolated, and polymer solution was added as described above. 100 µL of the erythrocyte-polymer suspension was transferred to a transparent flat-bottomed 96-well plate (VWR, Germany) in triplicate. The cells were incubated at 37 °C for 2 h, and the absorbance was measured at λ = 645 nm. Cells treated with PBS were the negative control, and cells treated with 50 µg mL^{-1} of 25 kDa LPEI were the positive control. The results are shown in **Figure S19**. The aggregation potential of the polymers was calculated as follows:

$$\text{Aggregation} = \frac{A_{Negative\ control}}{A_{Sample}} \quad \textbf{(S6)}$$

Where A_{Sample} and $A_{Negative\ control}$ are the absorption values of a given sample and the PBS treatment, respectively. Experiments were run in technical triplicates with blood from three blood donors.

1.12 Statistics

All assays were made at least in triplicate. Statistical analyses were calculated using one-way analysis of variance (ANOVA) by OriginPro2022b software. Additionally, if a statistically significant difference is given ($p \leq 0.05$), Bonferroni's posthoc test was performed. Statistical significance was notated as $*p \leq 0.05$,$**p \leq 0.01$, and $***p < 0.001$.

2. Results

2.1 Characterization of monomers by NMR

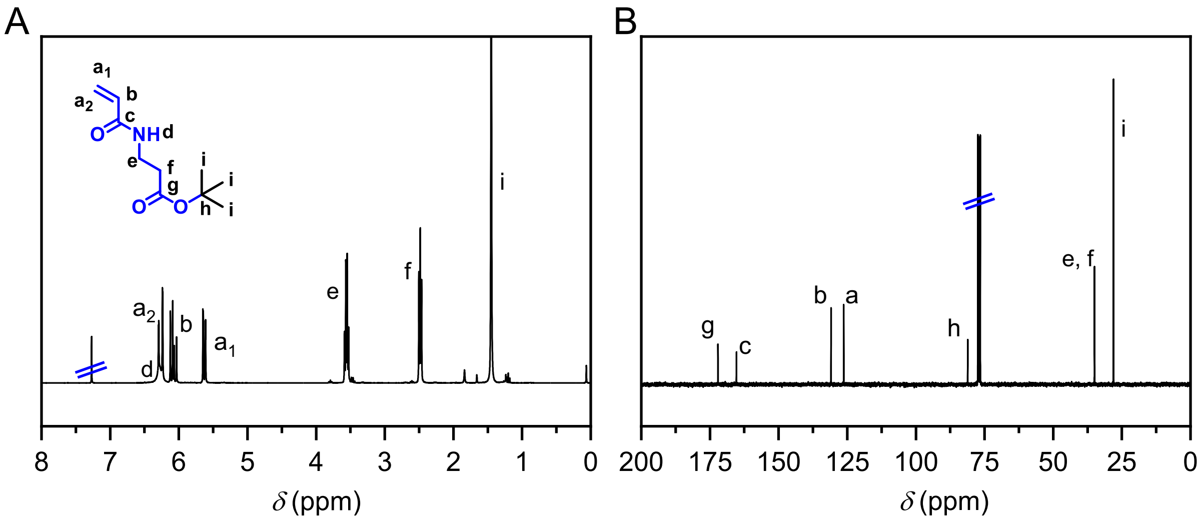

Figure S2. A ^{1}H NMR and **B** ^{13}C NMR spectra of CEAm^{tB} in $CDCl_3$ (300 MHz).

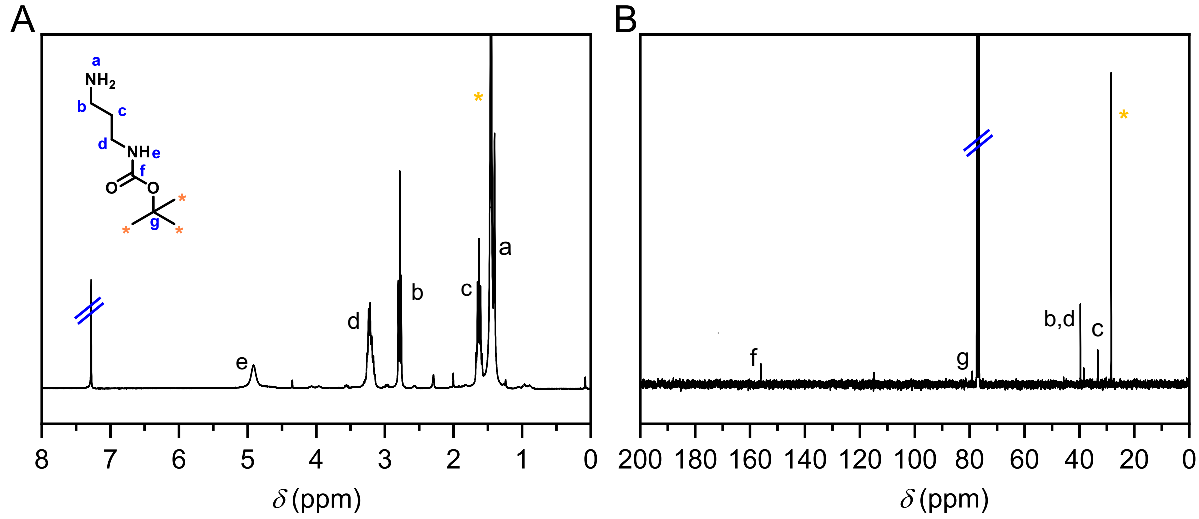

Figure S3. A ^{1}H NMR and **B** ^{13}C NMR spectra of *N*-*tert*-butoxycarbonyl-1,2-diaminopropane **(1)** in $CDCl_3$ (300 MHz).

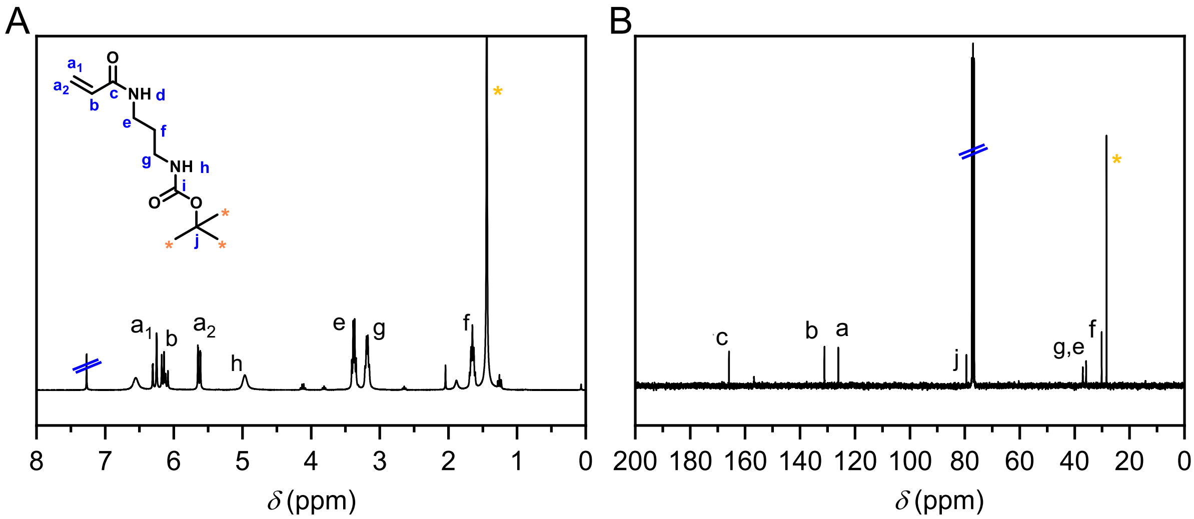

Figure S4. **A** ^{1}H NMR and **B** ^{13}C NMR spectra of *N-tert*-butoxycarbonyl-*N'*-aryloyl-1,2-diaminopropane **(2)** in $CDCl_3$ (300 MHz).

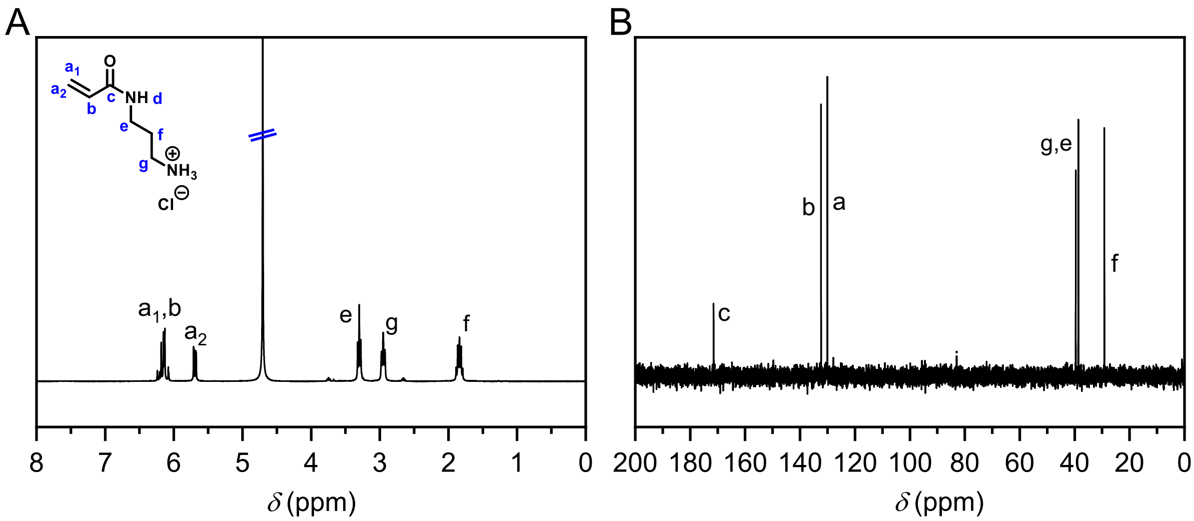

Figure S5. **A** ^{1}H NMR and **B** ^{13}C NMR spectra of *N*-acryloyl-1,3-diaminopropane hydrochloride **(3)** in D_2O (300 MHz).

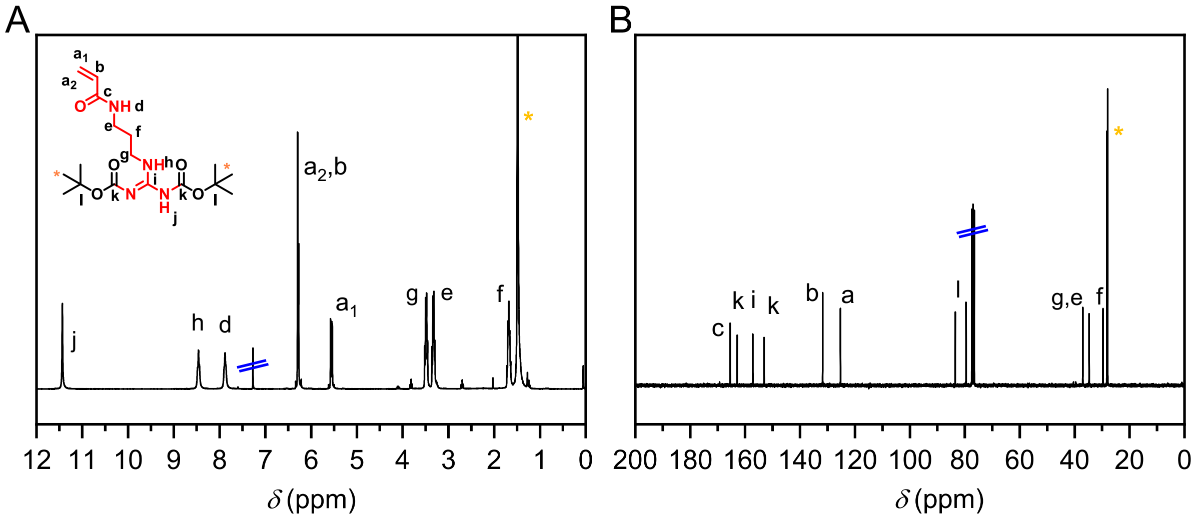

Figure S6. **A** ^{1}H NMR and **B** ^{13}C NMR spectra of $GPAm^{diBoc}$ in $CDCl_3$ (300 MHz).

2.2 Characterization of polymers by SEC

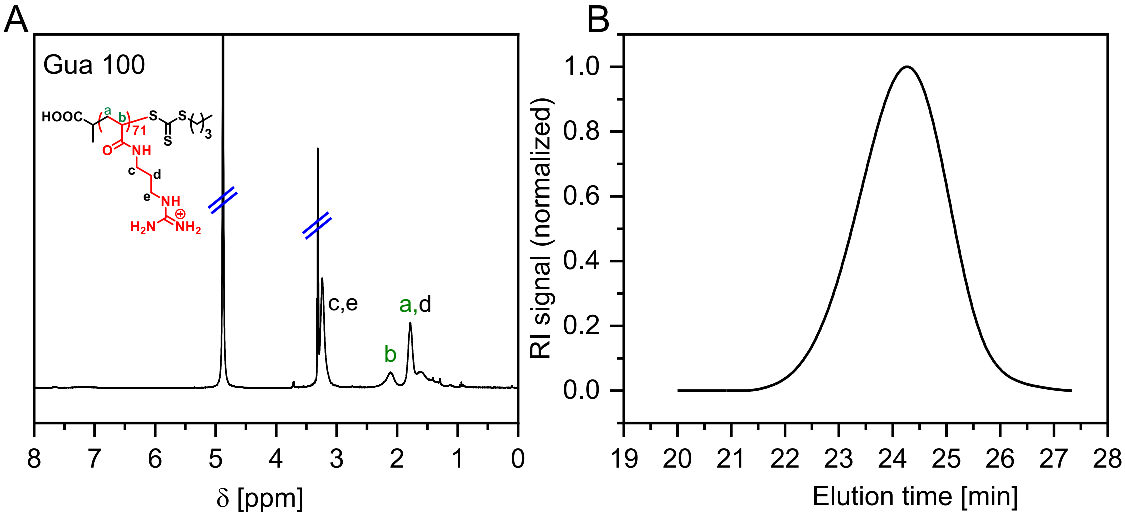

Figure S7. Characterization of Gua 100 (P(GPAm)$_{71}$). **A** ^{1}H NMR spectrum in CD_3OD (300 MHz); **B** Acidic SEC trace (eluent: 0.1 M NaCl + 0.1 wt.% TFA; calibration: Poly(2-vinylpyridine); $M_{n,SEC}$ = 7.5 kg mol^{-1}, $Đ$ = 1.44).

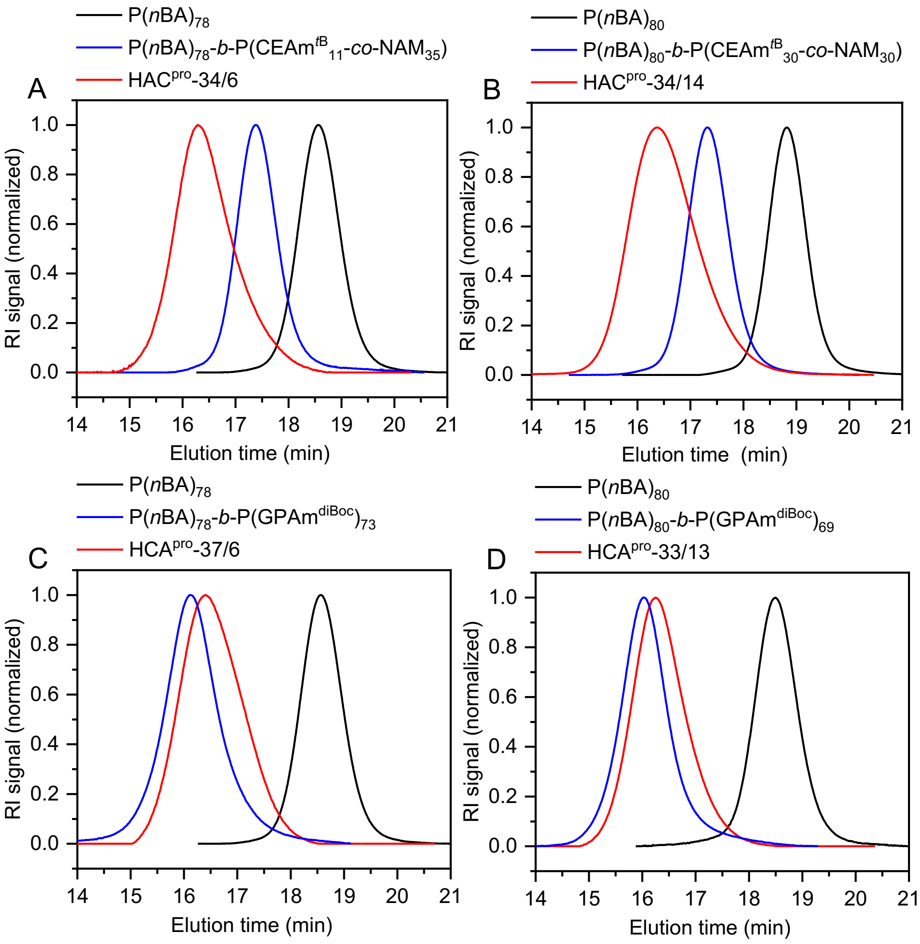

Figure S8. SEC traces of P(nBA)$_x$, precursors and **HAC^{pro}-34/6 A**, **-34/14 B**, and **HCA^{pro}-37/6 C** and **-33/13 D** using (eluent: DMAc + 0.21% LiCl, PMMA-calibration).

Table S6. Overview of the composition and characterization of P(*n*BA) (first blocks) and the diblock copolymers (second blocks).

**Composition**[a]	$M_{\mathrm{n,theo}}$[b] [kg mol^{-1}]	$M_{\mathrm{n,SEC}}$[c] [kg mol^{-1}]	*Đ*[c]
P(*n*BA)$_{78}$	10.2	11.4	1.07
P(*n*BA)$_{80}$ (1)	10.5	11.6	1.07
P(*n*BA)$_{80}$ (2)	10.5	11.9	1.09
P[(*n*BA)$_{78}$-*b*-(CEAm$^{\mathrm{tB}}_{11}$-*co*-NAM$_{35}$)]	17.4	22.6	1.10
P[(*n*BA)$_{78}$-*b*-(CEAm$^{\mathrm{tB}}_{17}$-*co*-NAM$_{37}$)]	18.9	25.9	1.07
P[(*n*BA)$_{80}$-*b*-(CEAm$^{\mathrm{tB}}_{30}$-*co*-NAM$_{30}$)]	20.7	28.9	1.09
P[(*n*BA)$_{78}$-*b*-(GPAm^{diBoc})$_{73}$]	37.3	40.0	1.18
P[(*n*BA)$_{78}$-*b*-(GPAm^{diBoc})$_{61}$]	32.8	33.2	1.24
P[(*n*BA)$_{80}$-*b*-(GPAm^{diBoc})$_{69}$]	36.1	46.5	1.15

[a] Numbers were determined *via* ^{1}H NMR spectroscopy and represent the DP of each monomer; [b] calculated using **Equation S2**; [c] determined *via* SEC (eluent: DMAc + 0.21% LiCl; PMMA standard).

2.3 Kinetic studies

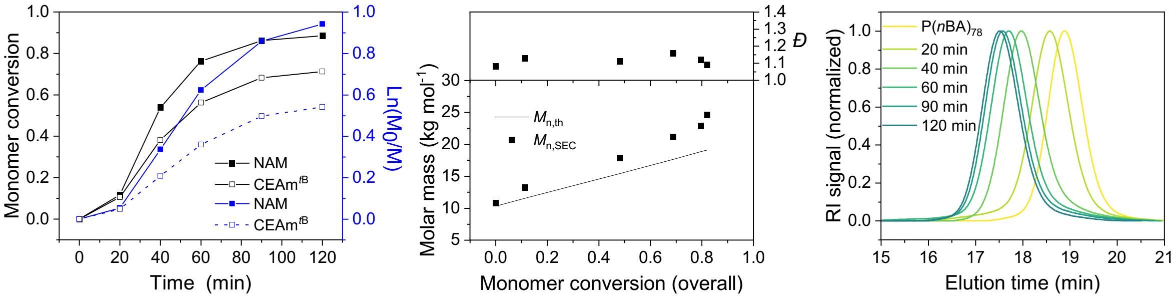

Figure S9. Kinetic study of the chain extension of P(*n*BA)$_{78}$ by RAFT polymerization of NAM and CEAm$^{t\mathrm{B}}$, obtaining P[(*n*BA)$_{78}$-*b*-(CEAm$^{\mathrm{tB}}_{17}$-*co*-NAM$_{37}$)]. Plot of the monomer conversion and ln(M_0/M) *vs.* time (**left**); plot of the molar mass and dispersity *vs.* the overall monomer conversion (**middle**); plot of the SEC traces (eluent: DMAc + 0.21 wt% LiCl, PMMA-calibration) (**right**).

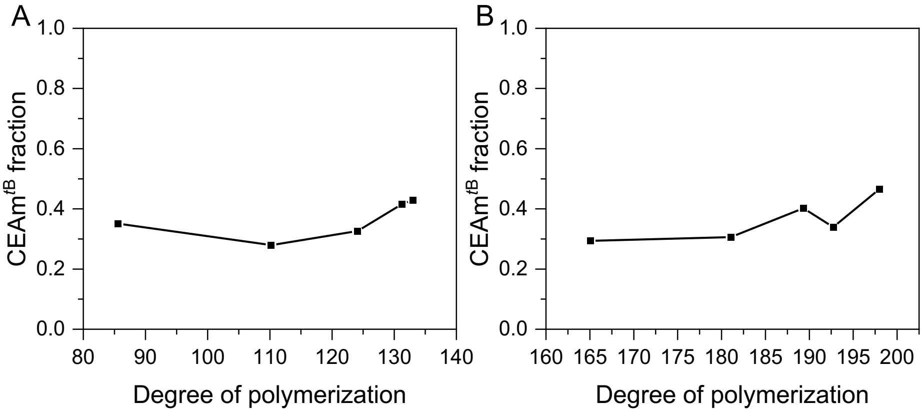

Figure S10. Incorporated fraction of CEAm$^{t\mathrm{B}}$ in the second block of P[(*n*BA)$_{78}$-*b*-(CEAm$^{\mathrm{tB}}_{17}$-*co*-NAM$_{37}$)] **A** and in the third block of BGCN (P[(*n*BA)$_{78}$-*b*-(GPAm^{diBoc})$_{59}$-*b*-(CEAm$^{t\mathrm{B}}_{20}$-*co*-NAM$_{40}$)]) **B**.

For the kinetic study of the polymerization of **HCA^{pro}**, approximately the same molar amount of CEAm^{tB} and NAM were used as for **HCA^{pro}**-31/9.

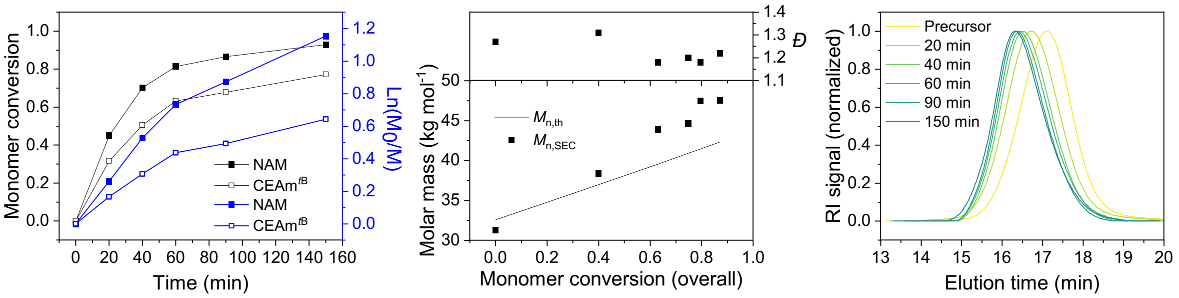

Figure S11. Kinetic study of the chain extension of P[(nBA)$_{78}$-b-(GPAm^{diBoc})$_{59}$] (precursor)by RAFT polymerization of NAM and CEAm^{tB}, obtaining P[(nBA)$_{78}$-b-(GPAm^{diBoc})$_{59}$-b-(CEAm^{tB}$_{20}$-co-NAM$_{40}$)]. Plot of the monomer conversion and ln(M$_0$/M) *vs.* time (**left**); plot of the molar mass and dispersity *vs.* the overall monomer conversion (**middle**); plot of the SEC traces, using (DMAc + 0.21 wt% LiCl)-SEC with PMMA-calibration (**right**).

2.4 Characterization of polymers by NMR

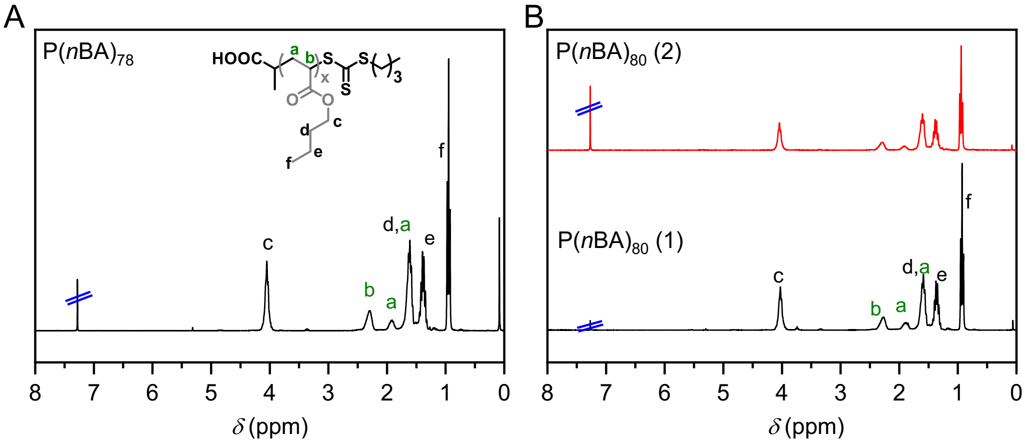

Figure S12. ^{1}H NMR spectra of P(nBA)$_{78}$ **A** and P(nBA)$_{80}$ (1) and (2) **B** in $CDCl_3$ (300 MHz).

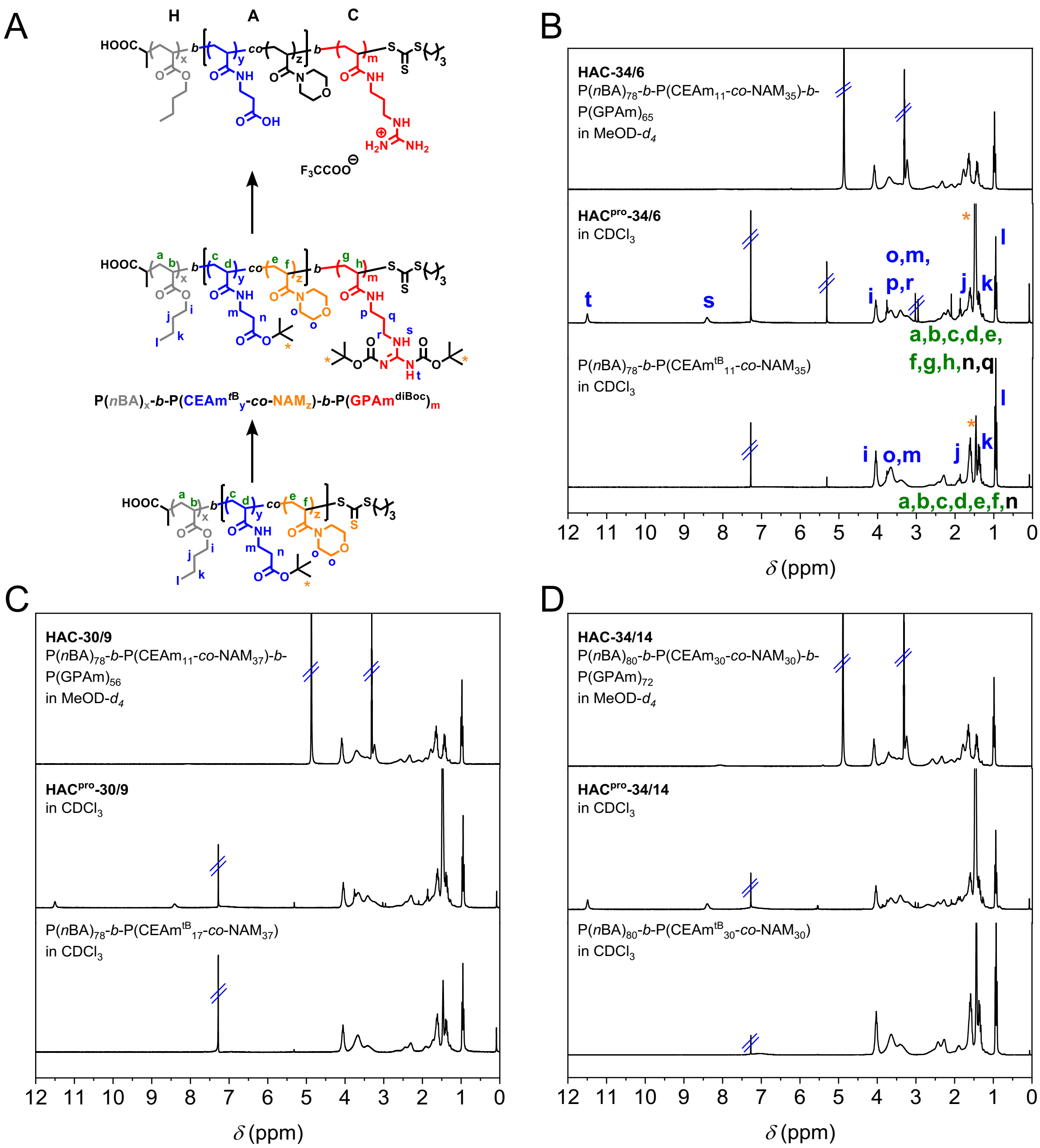

Figure S13. A Reaction scheme for chain extension of P(nBA)$_x$-b-P(CEAm$^{tB}{}_y$-co-NAM$_z$) to obtain **HAC^{pro}**, followed by deprotection to obtain **HAC**. ^{1}H NMR spectra of precursor polymers and **HAC-34/6 B**, **-30/9 C** and **-34/14 D** (300 MHz).

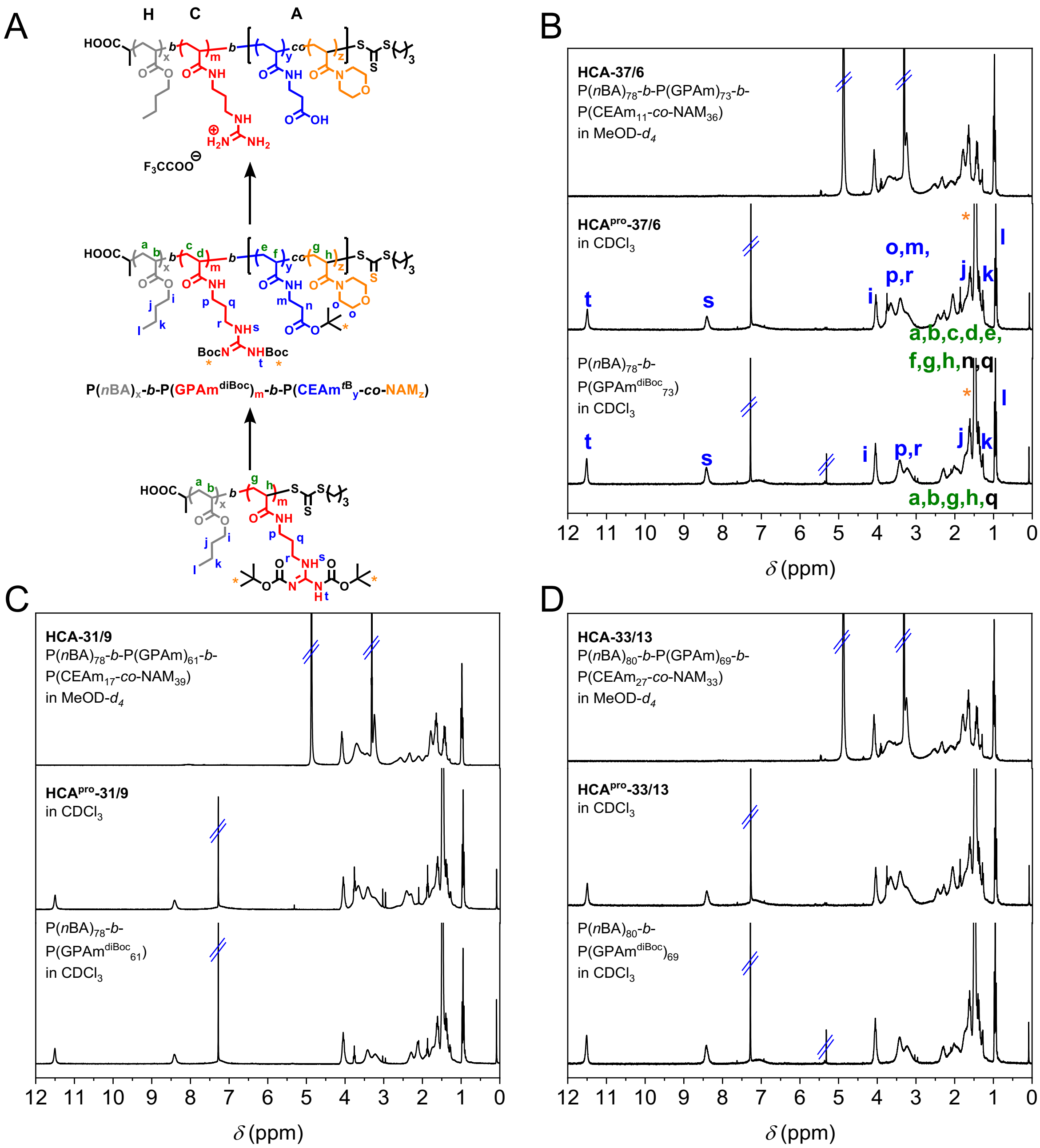

Figure S14. A Reaction scheme for the chain extension of P(nBA)$_x$-b-P(GPAm$^{diBoc}{}_m$) to obtain **HCA^{pro}**, followed by deprotection to obtain **HCA**. ^{1}H NMR spectra of precursor polymers and **HCA-37/6 B**, **-31/9 C** and **-33/13 D** (300 MHz).

2.5 Characterization of triblock micelles and micelleplexes by DLS & ELS

Intensity-, volume- and number-weighted size distributions and exponential decay correlation coefficients are shown in triplicates (left to right).

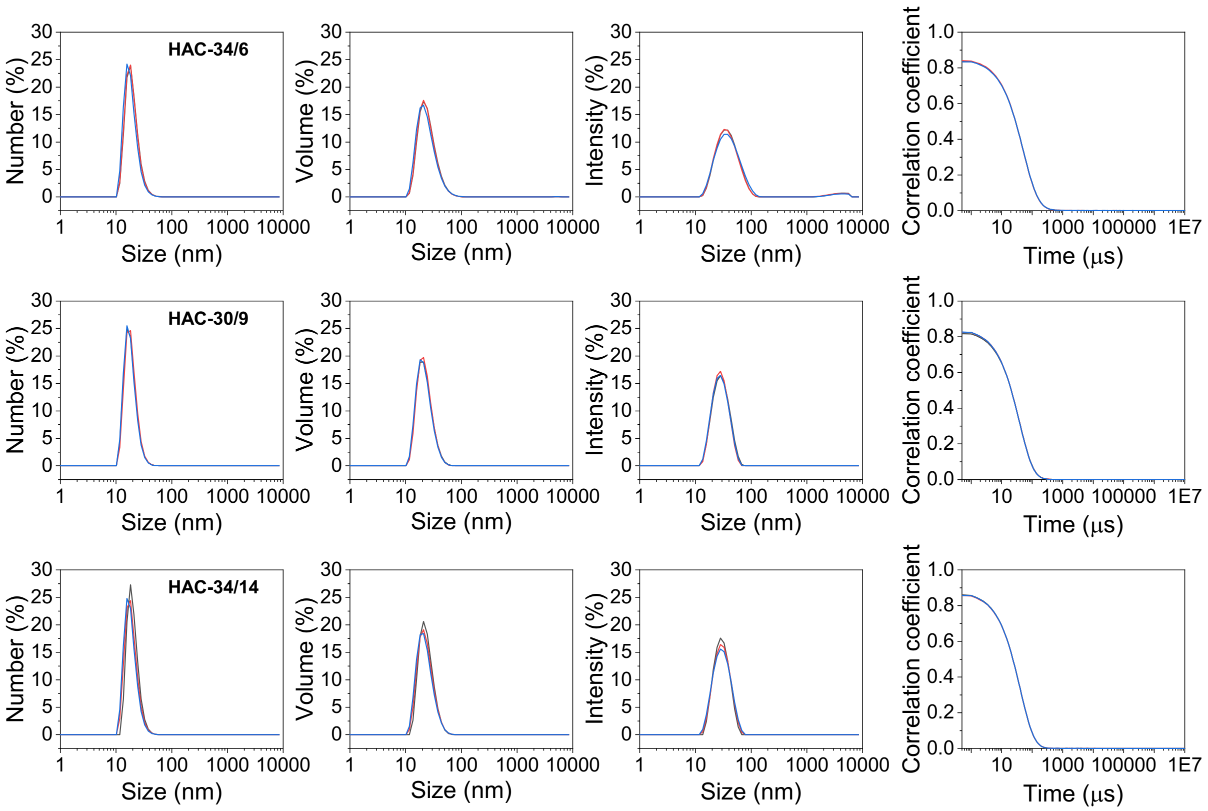

Figure S15. Size measurements of **HAC** micelles by DLS.

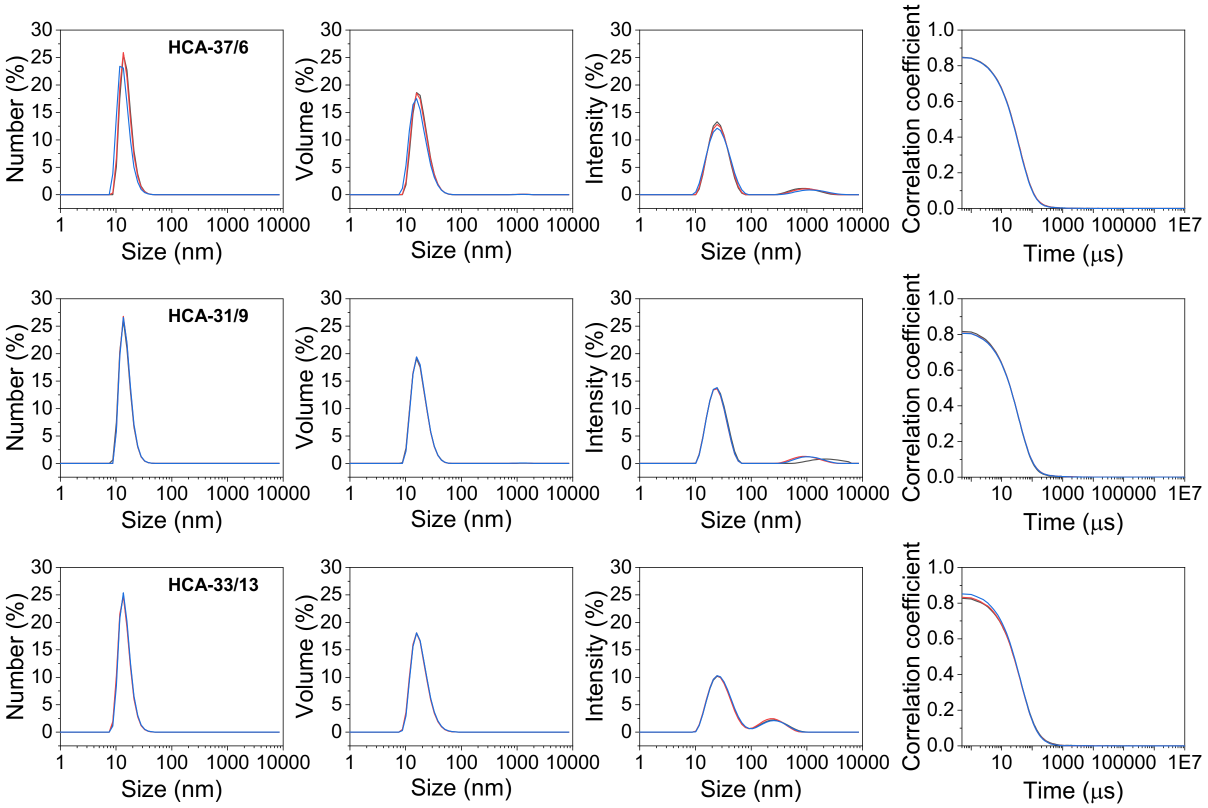

Figure S16. Size measurements of **HCA** micelles by DLS.

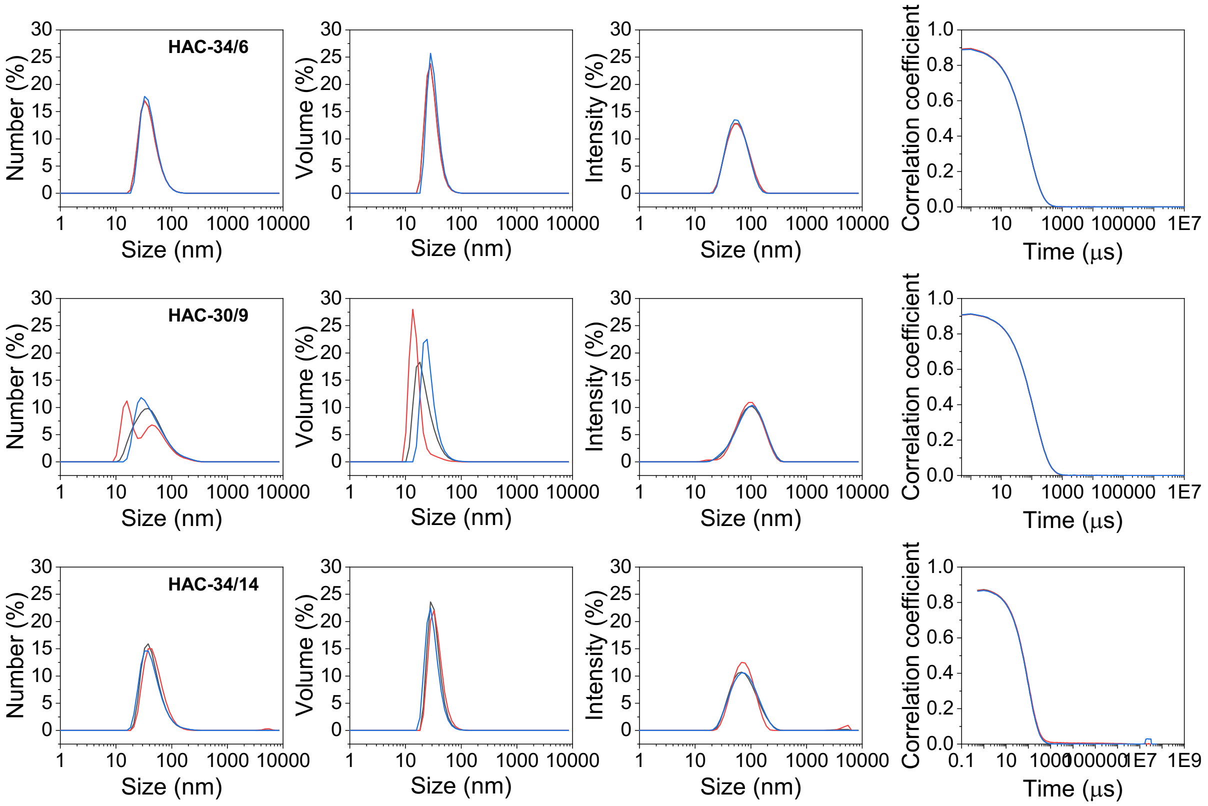

Figure S17. Size measurements of **HAC** micelleplexes by DLS.

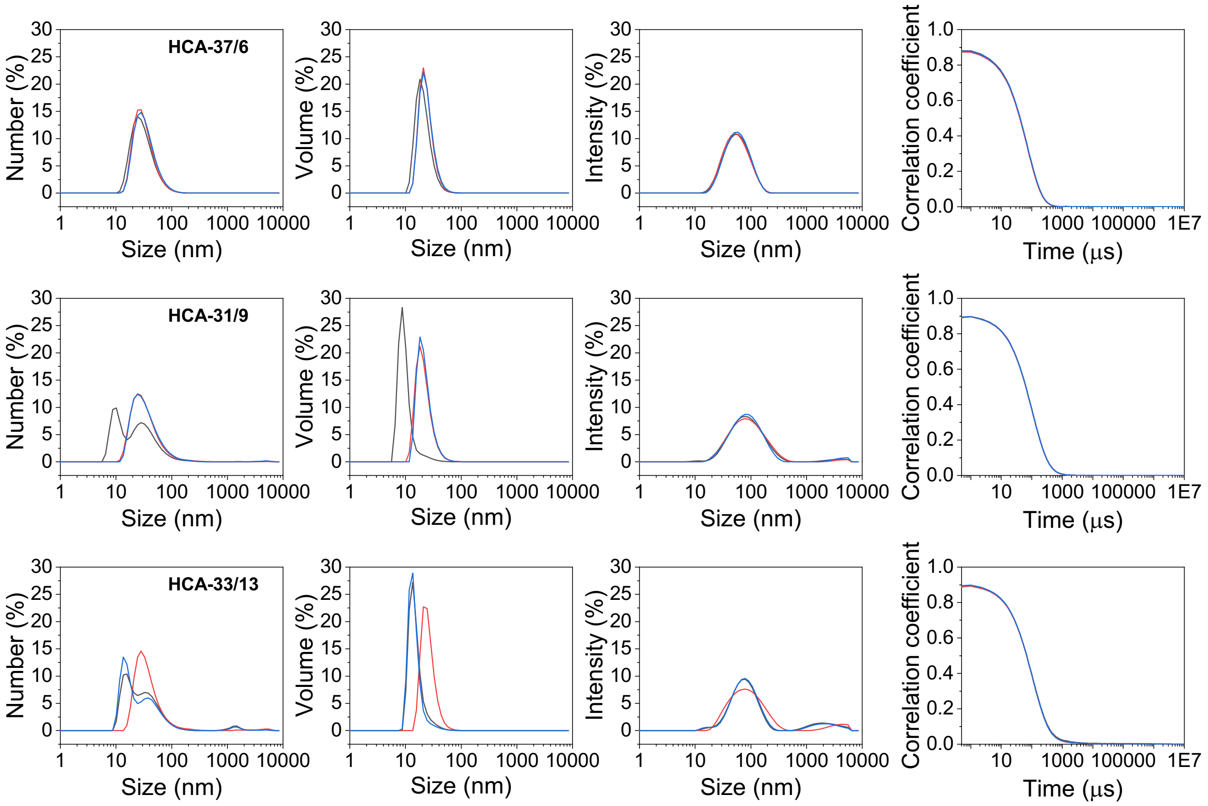

Figure S18. Size measurements of **HCA** micelleplexes by DLS.

2.6 Determination of metabolic activity in L929 cells (PrestoBlueassay)

Table S7. Polymer concentration used for transfection efficiency assay and LC_{50} of PrestoBlue assay. LC_{50} and relative metabolic activity of L929 cells at micelleplex concentration used for transfection efficiency assay calculation were done with DoseRespond fit function using OriginPro Software (Version 2022b).

Polymer ID	**LPEI**	**Gua 100**	**HAC-34/6**	**HCA-37/6**	**HAC-30/9**	**HCA-31/9**	**HAC-34/14**	**HCA-33/13**
LC_{50} / µg mL^{-1}	26.17	13.77	45.94	69.76	133.40	116.27	108.81	115.11
Micelleplex concentration on HEK93T cells/ (µg mL-1)	4	16	39	37	45	43	40	41
Relative metabolic activity of L929 cells at micelleplex concentration used for transfection efficiency assay (%)	95	43	60	79	99	83	96	89

2.7 Investigation of uptake and endosomal release

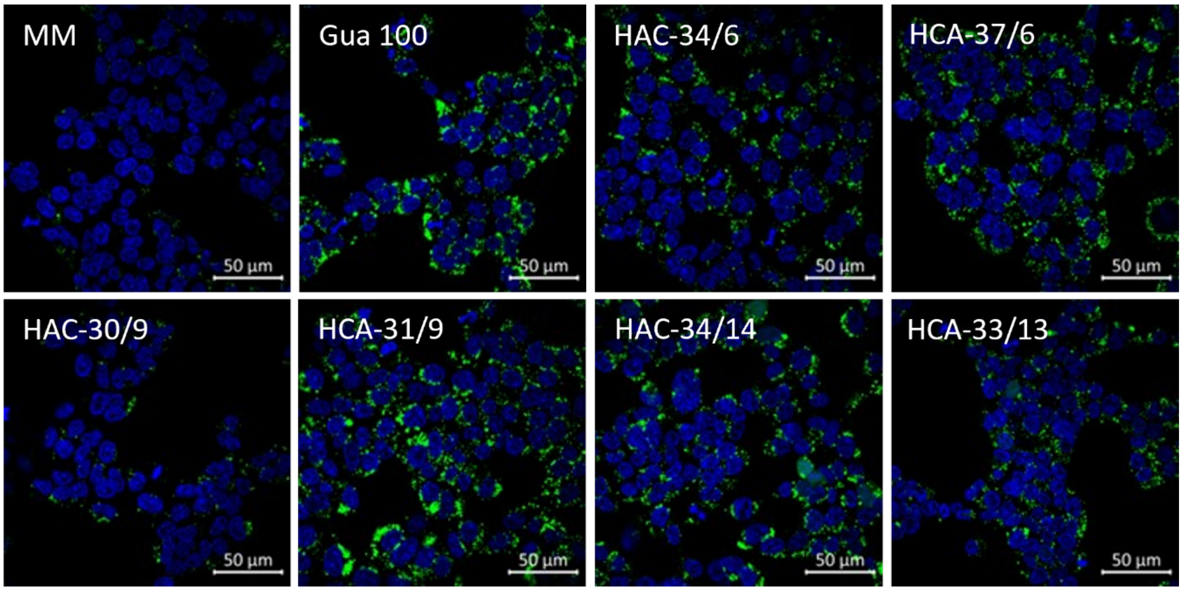

Figure S19. Endosomal release was analyzed via confocal laser scanning microscopy (CLSM). HEK293T cells were simultaneously incubated with the non-permeable dye calcein (green) and micelleplexes with N*/P 20, a pDNA concentration of 3 µg mL-1 on cells over 6 h incubation in D10H (6 h). The cell nuclei were stained with Hoechst 33342 (blue). Green dots in indicate endocytotic uptake of calcein within cellular compartments, and diffuse green fluorescence pattern indicates calcein released to the cytosol. pDNA-master mix-treated cells were used as the control (MM).

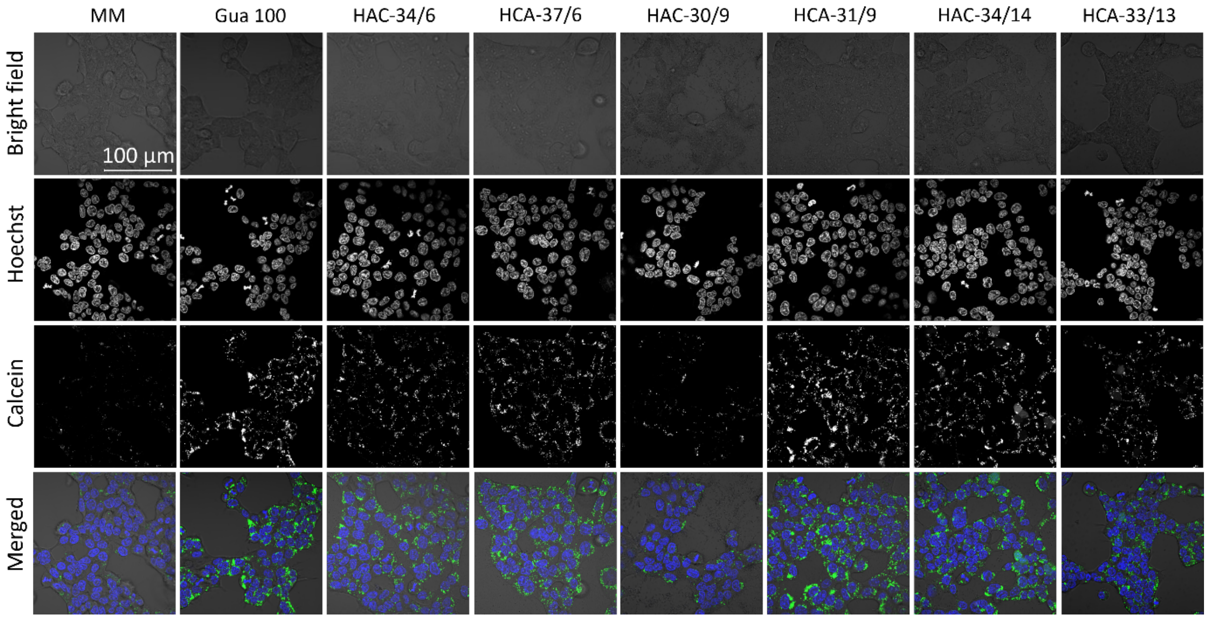

Figure S20. Gray images of HEK293T cells for endosomal release investigation. HEK293T cells were simultaneously incubated with the non-permeable dye calcein (final concentration of 25 µg mL-1) and micelleplexes with N*/P 20, a pDNA concentration of 3 µg mL-1 on cells over 6 h incubation in D10H (6 h). The cell nuclei were stained with Hoechst 33342. Calcein dots indicate endocytotic uptake of calcein within cellular compartments, and diffuse fluorescence pattern indicates calcein released to the cytosol. pDNA-master mix-treated cells were used as the control (MM).

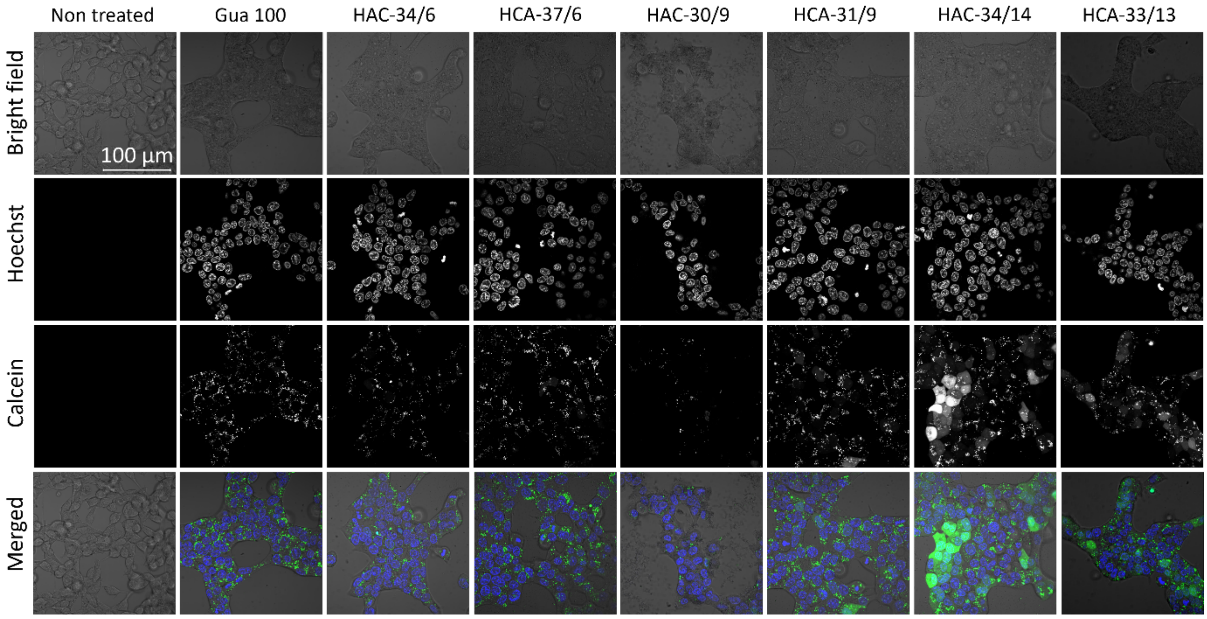

Figure S21. Gray images of HEK293T cells for endosomal release investigation. HEK293T cells were simultaneously incubated with the non-permeable dye calcein (final concentration of 25 µg mL-1) and micelleplexes with N*/P 20 with a pDNA concentration of 3 µg mL-1 on cells over 6 h incubation in D10H (6 h). Afterwards cells were further incubated for 2 h in D20 (6+2 h). The cell nuclei were stained with Hoechst 33342. Calcein dots indicate endocytotic uptake of calcein within cellular compartments, and diffuse fluorescence pattern indicates calcein released to the cytosol. pDNA-master mix-treated cells were used as the control (MM). Colored images of the calcein and the Hoechst 33342 channels can be found in the main article.

2.8 Erythrocyte aggregation and hemolysis

The **HCA**-micelle revealed in principle a higher aggregation rate at pH 6, which intensified by increasing cationic and likewise, anionic moieties. At pH 7, where the anionic moiety is less charged, the aggregation is more dependent on the content of cationic moieties.

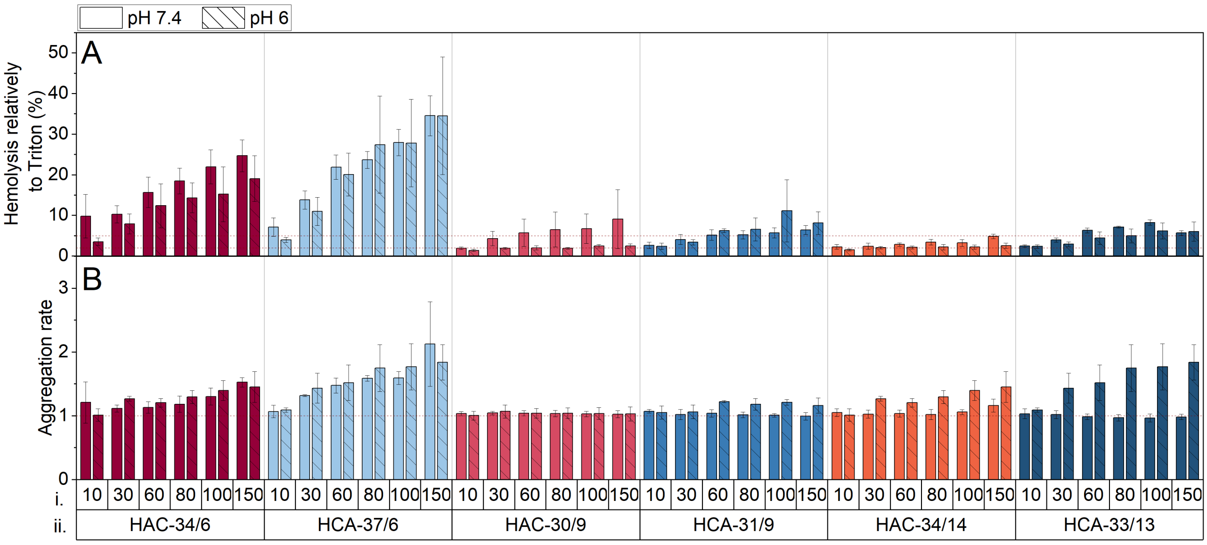

Figure S22. Hemolysis and aggregation assay of the mixed micelles. **A** Hemolysis assay was performed at pH 7.4 and pH 6 to investigate the polymer-cell membrane- and polymer-endosomal membrane-interaction. The stronger the interaction, the higher the amount of released hemoglobin calculated relative to the positive control Triton X-100. Values represent the mean ± SD (n = 3) of three donors. Hemolysis between 2% and 5% is considered non-hemolytic and slightly hemolytic, respectively. **B** Aggregation assay was performed at pH 7.4 and pH 6 i. Micelles concentration (µg mL^{-1}), ii. Micelles code.

References

1. Larnaudie, S. C.; Brendel, J. C.; Jolliffe, K. A.; Perrier, S. Cyclic peptide-polymer conjugates: Grafting-to vs grafting-from. *J. Polym. Sci., Part A: Polym. Chem.* **2016**, *54* (7), 1003-1011. DOI: 10.1002/pola.27937
2. Leer, K.; Reichel, L. S.; Kimmig, J.; Richter, F.; Hoeppener, S.; Brendel, J. C.; Zechel, S.; Schubert, U. S.; Traeger, A. Optimization of Mixed Micelles Based on Oppositely Charged Block Copolymers by Machine Learning for Application in Gene Delivery. *Small* **2023**, e2306116. DOI: 10.1002/smll.202306116
3. Hobson, L. J.; Feast, W. J. Poly(amidoamine) hyperbranched systems: synthesis, structure and characterization. *Polymer* **1999**, *40* (5), 1279-1297. DOI: 10.1016/S0032-3861(98)00268-7
4. Nizalapur, S.; Kimyon, O.; Yee, E.; Ho, K.; Berry, T.; Manefield, M.; Cranfield, C. G.; Willcox, M.; Black, D. S.; Kumar, N. Amphipathic guanidine-embedded glyoxamide-based peptidomimetics as novel antibacterial agents and biofilm disruptors. *Org. Biomol. Chem.* **2017**, *15* (9), 2033-2051. DOI: 10.1039/c7ob00053g
5. Richter, F.; Martin, L.; Leer, K.; Moek, E.; Hausig, F.; Brendel, J. C.; Traeger, A. Tuning of endosomal escape and gene expression by functional groups, molecular weight and transfection medium: a structure-activity relationship study. *J. Mater. Chem. B* **2020**, *8* (23), 5026-5041. DOI: 10.1039/d0tb00340a
